# Tracking the processing of damaged DNA double-strand break ends by ligation-mediated PCR: increased persistence of 3′-phosphoglycolate termini in SCAN1 cells

**DOI:** 10.1093/nar/gkt1347

**Published:** 2013-12-25

**Authors:** Konstantin Akopiants, Susovan Mohapatra, Vijay Menon, Tong Zhou, Kristoffer Valerie, Lawrence F. Povirk

**Affiliations:** ^1^Department of Pharmacology and Toxicology, Massey Cancer Center, Virginia Commonwealth University, Richmond, VA 23298, USA and ^2^Department of Radiation Oncology, Massey Cancer Center, Virginia Commonwealth University, Richmond, VA 23298, USA

## Abstract

To track the processing of damaged DNA double-strand break (DSB) ends *in vivo*, a method was devised for quantitative measurement of 3′-phosphoglycolate (PG) termini on DSBs induced by the non-protein chromophore of neocarzinostatin (NCS-C) in the human Alu repeat. Following exposure of cells to NCS-C, DNA was isolated, and labile lesions were chemically stabilized. All 3′-phosphate and 3′-hydroxyl ends were enzymatically capped with dideoxy termini, whereas 3′-PG ends were rendered ligatable, linked to an anchor, and quantified by real-time Taqman polymerase chain reaction. Using this assay and variations thereof, 3′-PG and 3′-phosphate termini on 1-base 3′ overhangs of NCS-C-induced DSBs were readily detected in DNA from the treated lymphoblastoid cells, and both were largely eliminated from cellular DNA within 1 h. However, the 3′-PG termini were processed more slowly than 3′-phosphate termini, and were more persistent in tyrosyl-DNA phosphodiesterase 1-mutant SCAN1 than in normal cells, suggesting a significant role for tyrosyl-DNA phosphodiesterase 1 in removing 3′-PG blocking groups for DSB repair. DSBs with 3′-hydroxyl termini, which are not directly induced by NCS-C, were formed rapidly in cells, and largely eliminated by further processing within 1 h, both in Alu repeats and in heterochromatic α-satellite DNA. Moreover, absence of DNA-PK in M059J cells appeared to accelerate resolution of 3′-PG ends.

## INTRODUCTION

Most double-strand breaks (DSBs) induced in a natural environment or as a result of chemotherapy do not bear ligatable 3′**-**hydroxyl and 5′**-**phosphate termini, but instead are blocked at one or both ends by covalently bound proteins or fragmented nucleotides that must be removed before the breaks can be resealed. Although several enzymes capable of removing terminal blocks have been identified (1,2), in many cases it is still unclear which of them are actually used for the repair of various types of DSBs *in vivo*. Radiomimetic natural products, including bleomycin, neocarzinostatin and calicheamicin, each induce DSBs by site-specific free radical attack on deoxyribose. Although the ends of these DSBs are not homogeneous, the chemical structure of the termini as well as the stagger between breaks in opposite strands is limited to one or a few possibilities, and for all three agents, a substantial fraction of the DSBs bear 3′-phosphoglycolate (3′-PG) termini (3,4). Biochemical studies indicate that several enzymes, including tyrosyl-DNA phosphodiesterase 1 (TDP1) (5) apurinic/apyrimidinic endonuclease 1 (APE1) (6) and Artemis nuclease (7), are capable of resolving these lesions. APE1 has been implicated in repair of bleomycin-induced DSBs (8), whereas cytogenetic and survival assays suggest a role for TDP1 in resolution of 3′-PG termini of calicheamicin-induced DSBs (9). In the case of topoisomerase II-mediated DSBs, survival and focus-formation assays suggest that TDP2 and CtIP are each involved in separate pathways for removal of the 5′-tyrosyl-linked TOP2 fragments at TOP2-mediated DSBs (10,11).

However, in all of these studies, a deficiency in removal of 5′- or 3′-blocking groups was inferred from survival and DSB rejoining data, rather than being directly measured. Tracking the processing of DSB ends is difficult because only a few DSBs (∼50–100) are sufficient to kill a cell, so that even at supralethal concentrations of DSB-inducing agents, there will be, on average, only one modified end in several megabases of DNA, essentially precluding chemical methods of detection. To more directly assess the processing of blocked DSB termini, a polymerase chain reaction (PCR)-based assay was developed to detect and quantify 3′-PG, 3′-phosphate and 3′-hydroxyl termini on DSBs induced by non-protein chromophore of neocarzinostatin (NCS-C) at a specific site in the human Alu repeat sequence. The results show that while most 3′-phosphate termini are removed within a few minutes, 3′-PG termini are processed more slowly, and their persistence is increased by a deficiency in TDP1.

## MATERIALS AND METHODS

### Cells

Epstein–Barr virus-transformed lymphoblastoid cells derived from spinocerebellar ataxia with axonal neuropathy (SCAN1) patients and from unaffected relatives were obtained from Dr James Lupski, Baylor School of Medicine (12). Cells were maintained in 25 or 75-cm^2^ tissue culture flasks (vertical position) in RPMI medium plus 10% fetal bovine serum. For cell cycle studies, cells were diluted to 2.5 × 10^5^ cells/ml, and 3-ml cultures were grown overnight before addition of 1-nM neocarzinostatin and incubation for 12–48 h. Cells were stained with propidium iodide and subjected to cytometry as described (13). M059J and M059K glioma cells (14) (from Joan Turner, Cross Cancer Institute) were grown in monolayer culture in 1:1 DMEM/F-12 medium plus 10% fetal bovine serum.

### NCS-C treatment of DNA and cells

NCS-C (70–100 μM in 80% methanol) was prepared as described previously (15). Human DNA from mammary epithelial cells (100 μg/ml) or a pCR2.1-based plasmid harboring the Alu sequence (16) (200 μg/ml) was treated with NCS-C in the presence of 50 mM HEPES-KOH, pH 8, 1 mM ethylenediaminetetraacetic acid (EDTA) and 5 mM glutathione. Reactions were prepared on ice, with NCS-C (diluted in 20 mM sodium citrate, pH 4/20% methanol) being added last, and were incubated at 22°C for 1 h. For verification of the NCS-C-induced DSB hotspot in Alu, a fragment consisting of Alu bp 206–283 and 5′-labeled at bp 206 was PCR-amplified from pBLUR8 (16), and 0.2 μg in 20 μl was similarly treated with 3 μM of NCS-C, followed by incubation with 25 μg/ml recombinant TDP1 (17) for 1 h at 37°C, inactivation at 65°C for 10 min, addition of 10 mM MgCl_2_ and 5 μg/ml polynucleotide kinase/phosphatase (PNKP, gift of Michael Weinfeld, Cross Cancer Institute) and incubation for 1 h. Samples were ethanol-precipitated and analyzed on a sequencing gel.

For treatment of cells, exponentially growing lymphoblastoid cell cultures (∼5 × 10^5^ cells/ml) were pelleted at 600×*g*, for 5 min, washed with phosphate-buffered saline (PBS) and suspended in PBS on ice at a concentration of 2 × 10^6^ cells/ml. NCS-C (70–100 μM) or vehicle, usually 4 μl, was placed in the bottom of a 0.5-ml microcentrifuge tube on ice. Cell suspension, typically 45–60 μl, was added and rapidly mixed with the NCS-C by pipetting, then placed on ice for 5 min and in a 37°C water bath for 10 min (or, where indicated, in a 22°C heat block). Aliquots (usually 16 μl) of the cell suspensions were then either flash-frozen in liquid nitrogen, or diluted into 80 volumes of complete medium and incubated for various times to allow repair, after which the cells were pelleted at 14 000×*g*, decanted and flash-frozen. In some experiments, the DNA-dependent protein kinase (DNA-PK) inhibitor KU-57788 was added to cells from a 2.5-mM stock in DMSO, beginning 1 h before NCS-C treatment, and the inhibitor was present throughout washing, treatment and incubation. For treatment of M059J and M059K cells, cultures were trypsinized, washed and resuspended, and then treated and harvested as above, except that attached cells were released by trypsinization following repair incubations.

### Detection of DSB ends by Taqman PCR

After all samples were harvested, they were stored at −80° for ≤24 h and then resuspended in 200 μl of PBS. DNA was isolated from the cells using Qiagen DNeasy columns according to the manufacturer’s instructions, with a volume of 200 μl for the final elution step. DNA was ethanol-precipitated in the presence of 0.3 M NaOAc and 1 μg tRNA. The DNA was dissolved in 52 μl of 10 mM Tris–HCl, pH 8/0.1 mM EDTA (TE), and 28 μl of 1 M Tris–HCl, pH 7, was added. To reduce unstable NCS-C-induced 5′-aldehyde groups to 5′-hydroxyls, fresh 1 M NaBH_4_ was added in three aliquots of 6.7 μl, with a 30-min incubation at 22°C following each addition (18). The samples were diluted to 160 μl, and the DNA was ethanol-precipitated and then treated with 0.1 M putrescine in 0.1 M HEPES–KOH, pH 8/2 mM EDTA in a volume of 40 μl, to cleave abasic sites (19). For isolated DNA treated directly with NCS-C, these chemical treatments were similar, but DNA was not precipitated before NaBH_4_ addition.

DNA was again precipitated and then treated with 2.5 μg/ml PNKP for 2 h at 37°C in 20 μl of 1.2× terminal transferase (TdT) buffer [1× = 50 mM KOAc/20 mM Tris-acetate, pH 7.9/10 mM Mg(OAc)_2_] to remove 3′-phosphates. To render the resulting 3′-hydroxyl termini unligatable, 2.4 μl of 2.5 mM CoCl_2_, 0.6 μl of a 10 mM 40:1 mixture of dTTP and ddTTP and 10 U of TdT (New England Biolabs) were added, and the samples were incubated for 30 min at 37°C. After addition of 0.8 μl of 0.5 M EDTA, DNA was deproteinized by extracting with phenol, then with chloroform and precipitated. It was then dissolved in 20 μl of 20 mM Tris–HCl, pH 7.6/0.1 mM EDTA/1 mM dithiothreitol/50 μg/ml bovine serum albumin, divided into two equal aliquots and treated (or not) with 50 μg/ml of recombinant human TDP1 (17) for 2 h at 37°C to convert 3′-PG to 3′-phosphate termini. TDP1 was inactivated by heating to 65°C for 10 min, and then NEB buffer 3 (100 mM NaCl/50 mM Tris–HCl, pH 7.9/10 mM MgCl_2_/1 mM dithiothreitol) and 300 U/ml of calf intestinal phosphatase (CIP) were added. Samples were incubated for 30 min at 37°C to remove all 3′- and 5′-phosphates. Samples were diluted to 20 μl, again extracted with phenol and chloroform, precipitated and dissolved in 20 μl of TE. The DNA concentration of each sample was determined by real-time PCR of Alu repeats (20) (Supplementary Figure S1).

For ligation, the anchor consisted of (5′-hydroxyl)-TGCCGATCATGCAACTCTGCGTCAAATCGCGATCAGTddC, where ddC indicates a 3′-terminal dideoxycytidine, annealed to (5′-phosphate)-ACTGATCGCGATTTGACGCAGAGTTGCATGATCGGCinvT (Integrated DNA technologies), where invT indicates an ‘inverted’ thymidine, linked to the penultimate nucleotide via a 3′-3′ phosphodiester bond. Ligation reactions were carried out at 16°C for 16 h and contained 5 ng of cellular DNA, 1.5 μM anchor and 180 cohesive-end units T4 DNA ligase (New England Biolabs) in 12 μl of 50 mM Tris–HCl, pH 7.5/10 mM MgCl_2_/1 mM ATP/10 mM dithiothreitol. Ligase was inactivated at 65°C for 10 min, and then each sample was diluted with water to a concentration of 50 ng/ml cellular DNA. The efficacy of all enzymatic manipulations was verified using an internally labeled plasmid bearing a 3′-PG-terminated 1-base 3′ overhang, constructed as described (5).

Real-time PCR reactions (20 μl) for DSB quantitation contained 1× Taqman Master Mix (Applied Biosystems), 1-μM forward Alu consensus primer GGCAGGAGTATCGCTTGAAC (Alu bp 186–205) or pBLUR8-specific Alu primer AGACAGAAGAATCCCTTAAACCAAGA, 1-μM reverse anchor primer TGCAACTCTGCGTCAAATCG, 250-nM Taqman probe 5′-(6-carboxyfluorescein)-AGGTTGCAGACTGATC-(MGBNFQ) where MGBNFQ is a minor groove-binding non-fluorescent quencher (Applied Biosystems) and 50-pg template DNA. Taqman PCR was conducted in an Applied Biosystems 9700HT with a thermal profile consisting of 10 min at 50°C and 5 min at 95°C, followed by 50 cycles of 15 s at 95°C and 1 min at 60°C. In later experiments, longer primers bearing 5′ extensions for high-throughput sequencing were used (Supplementary Table S1) because they unexpectedly yielded more robust Taqman PCR signals. Measurement of DSBs in the α-satellite sequence was performed similarly, except that the forward primer was GAGCAGTTTTGAAACACTCTTTTTGT [α-satellite consensus bp 56–90 (21)] and the probe was 5′-(6-carboxyfluorescein)-CTGCAAGACTGATCG-(MGBNFQ), for detection of DSBs at the AGT at position 103. Primers and probes were designed using PrimerExpress software (Applied Biosystems).

## RESULTS

### Principle of the 3′-PG DSB assay

Even in heavily damaged cells, DSBs are rare events and thus any assay for processing of DSB ends must be exquisitely sensitive. Real-time Taqman PCR is capable of detecting and quantifying as little as a single molecule of any sequence-specific DNA (22). Thus, in principle, even extremely rare lesions can be accurately measured, provided they occur at a specific sequence, and can be quantitatively converted to DNA breaks with ligatable ends.

**Figure 1.**
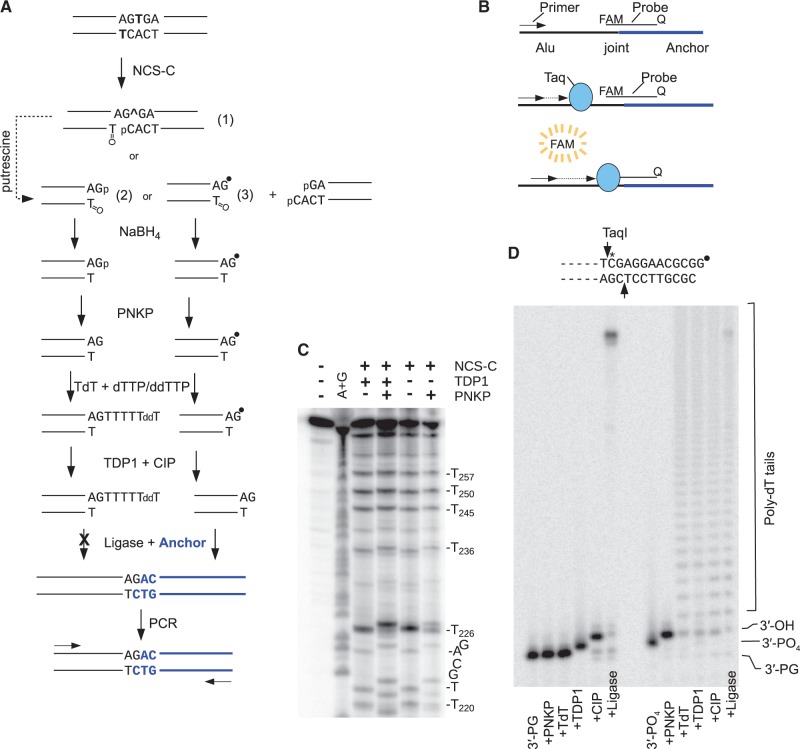
Principle of the Taqman PCR assay and verification of enzymatic manipulations. (**A**) Rationale. At AGT•ACT sites, NCS-C induces three types of bistranded lesions: (1) an abasic site (^) with a closely opposed break, (2) a DSB with a 3′-PG (•) terminus or (3) a DSB with a 3′-phosphate terminus. NaBH_4_ stabilizes the 5′-aldehyde, while putrescine converts abasic sites to strand breaks (dashed arrow). The resulting DSB ends are subjected to treatment with PNKP, TdT, TDP1 and CIP, such that 3′-phosphate DSBs are capped with unligatable tails, while 3′-PG DSBs are converted to 3′-hydroxyl DSBs, ligated to an anchor and amplified by PCR. (**B**) Quantitation by Taqman PCR. As PCR product accumulates, a fluorescent Taqman probe spanning the ligation site anneals to the joint sequence, and Taq polymerase releases the FAM fluorophore. (**C**) Verification of 3′-PG termini at the AGT sequence at Alu bp 224–226. An end-labeled fragment containing the BLUR8 Alu DNA clone was treated with NCS-C, and then treated with TDP1 and/or PNKP as indicated and analyzed on a denaturing sequencing gel. A substantial fraction of the band corresponding to a DSB at bp 226 required both TDP1 and PNKP to induce a shift to lower mobility, indicating the presence of DSBs with 3′-PG termini, and their conversion to 3-hydroxyl termini. (**D**) Verification that the enzymatic manipulation of DNA ends can be effected with near-quantitative efficiency. An internally labeled (asterisk) substrate containing a DSB with a one-base 3′ overhang and a 3′-PG or 3′-phosphate terminus (black circle) was treated successively with the indicated enzymes. A 12-base-labeled fragment was released from the 3′-end by TaqI and analyzed on a denaturing gel. The 3′-phosphate terminus is efficiently poly(dT)-tailed, while the 3′-PG terminus is converted to a 3′-hydroxyl and ligated to an oligomeric anchor duplex.

The antibiotic neocarzinostatin, produced by *Streptomyces carzinostaticus*, consists of a carrier protein plus a hydrophobic non-protein chromophore (NCS-C) that induces DSBs by concerted free radical attack on deoxyribose in both strands of DNA. Most NCS-C-induced lesions are single-strand breaks bearing 3′-phosphate and 5′-aldehyde termini, but ∼20% of these breaks are accompanied by a strand break or abasic site in the complementary strand, and AGT•ACT sequences are hotspots for these bistranded lesions (Figure 1A). In the ACT strand, T is oxidized to a 5′-aldehyde, producing a strand break. In the AGT strand, the T is oxidized to either an abasic site or a strand break bearing 5′-phosphate and either 3′-phosphate or 3′′-PG termini (23–25). Thus, the PG-terminated fraction of these lesions (∼25%) represents a possible probe for processing of protruding 3′-PG termini of DSBs, provided they can be distinguished from other lesions at the same site. An additional advantage of NCS-C is that it is activated by sulfhydryls in cells within minutes, and any that escapes activation rapidly degrades (26). Thus, damage can be induced at a defined time.

The human Alu sequence is repeated 1 million times throughout the human genome (27) and thus offers an attractive target for quantifying site-specific DSBs. For quantitation of NCS-C-induced DSBs, the AGT•ACT site at Alu bp 224–226 was chosen, allowing PCR primer positioning in a relatively well-conserved region at bp 176–205 (28).

### Assay development

To verify whether NCS-C induced 3′-PG-terminated breaks at the Alu bp 224–226 AGT•ACT site, the BLUR8 Alu sequence variant [clone 8 from (16)] was 5′-end-labeled and treated with NCS-C in the presence of glutathione. As predicted, denaturing gel electrophoresis indicated a strong cleavage site at the T at position 226 (Figure 1C). To determine the termini of these breaks, the sample was treated with TDP1, PNKP or both enzymes. PNKP treatment resulted in a small mobility shift for bands corresponding to most of the cleavage sites, presumably reflecting 3′-phosphate removal. However, at bp 226, only about half of the material in the band was shifted by PNKP alone, whereas most of the remainder was shifted following treatment with both TDP1 and PNKP. Because TDP1 converts 3′-PG to 3′-phosphate termini (5), these results confirm formation of 3′-PG breaks at this site, and previous results indicate that such breaks are almost exclusively DSBs (24,25).

Figure 1A shows the scheme for quantification of these 3′-PG DSBs. First, immediately after NCS-C treatment or isolation of DNA from treated cells, the unstable 5′-aldehyde groups are chemically reduced to 5′-hydroxyls with NaBH_4_ (18). Next, the DNA is treated with 0.1 M putrescine to cleave any abasic sites, converting them to 3′-phosphate-terminated strand breaks (19). At AGT•ACT sequences, the abasic sites induced by NCS-C are mostly C-4′-oxidized (25), and, in principle, NaBH_4_ treatment should stabilize these against amine-mediated cleavage (29). Nevertheless, there will likely be some C-1′-oxidized sites as well that will not be stabilized (30), and, in practice, the putrescine step was found to be essential for obtaining interpretable and reproducible data. These lesions, as well as the 3′-phosphate-terminated DSBs induced directly by NCS-C, are converted to 3′-hydroxyl DSBs by treatment with PNKP, and then rendered unligatable by addition of a dideoxy-terminated poly-dT tail with TdT plus a mixture of dTTP and ddTTP (40:1). Finally, the residual 3′-PG DSBs are converted to 3′-hydroxyl DSBs by treatment with TDP1 and CIP, ligated to a 5′-phosphate-terminated anchor, and quantified by Taqman real-time PCR, using a probe that spans the predicted joint between the anchor and the Alu sequence at the break (Figure 1B). The anchor has a ddC 3′ overhang, which can anneal to the predicted one-base 3′ overhang of the NCS-C-induced DSB, but cannot itself be ligated to any type of break. In addition, the anchor has unligatable 5′-hydroxyl and inverted nucleotide termini at the opposite end. Thus, in principle, the anchor can only be ligated to NCS-C-induced DSBs at which 3′-PG termini have been converted to 3′-hydroxyl termini, and only ligations with DSBs at the bp 224–226 hotspot will be detected by the Taqman probe.

To assess whether these enzymatic manipulations could be effected with the required near-quantitative efficiency, an internally labeled model DSB substrate bearing a 1-base PG-terminated 3′ overhang and a 5′-hydroxyl was constructed (Figure 1D). This substrate has a terminal structure identical to that of the NaBH_4_-reduced derivative of the NCS-C-induced DSBs that would be formed in cells. A corresponding 3′-phosphate-terminated DSB end was generated by treating this substrate with TDP1 (5). Each of these substrates was mixed with unlabeled DNA from NCS-C-treated cells so that overall concentrations of DNA and of DNA ends would match those of cell-based experiments (see later in the text), and then the mixtures were subjected to the same enzymatic manipulations to be used in those experiments. Aliquots were taken after each enzyme step, phenol-extracted and then treated with TaqI to release the labeled terminal fragment, which was denatured and analyzed on a sequencing gel.

As expected (Figure 1D), the 3′-PG terminus was unaffected by PNKP and TdT treatments, but was quantitatively converted to a 3′-hydroxyl by TDP1/CIP treatment, and the resulting substrate was efficiently ligated to the 5′-phosphate anchor. In contrast, the 5′-phosphate terminus was quantitatively converted to a 5′-hydroxyl by PNKP and then was efficiently poly(dT)-tailed by TdT, so that only a trace of apparent ligation product was detected, ∼10-fold less than for the 3′-hydroxyl terminus. This residual ligation may arise in part from a small fraction of 3′-hydroxyl termini that are lengthened by a single ddT nucleotide that is then removed by the weak 3′-nucleosidase activity of TDP1 (31). Nevertheless, these data indicate that the assay should provide an accurate measure of NCS-C-induced DSBs bearing 3′-PG termini.

**Figure 2.**
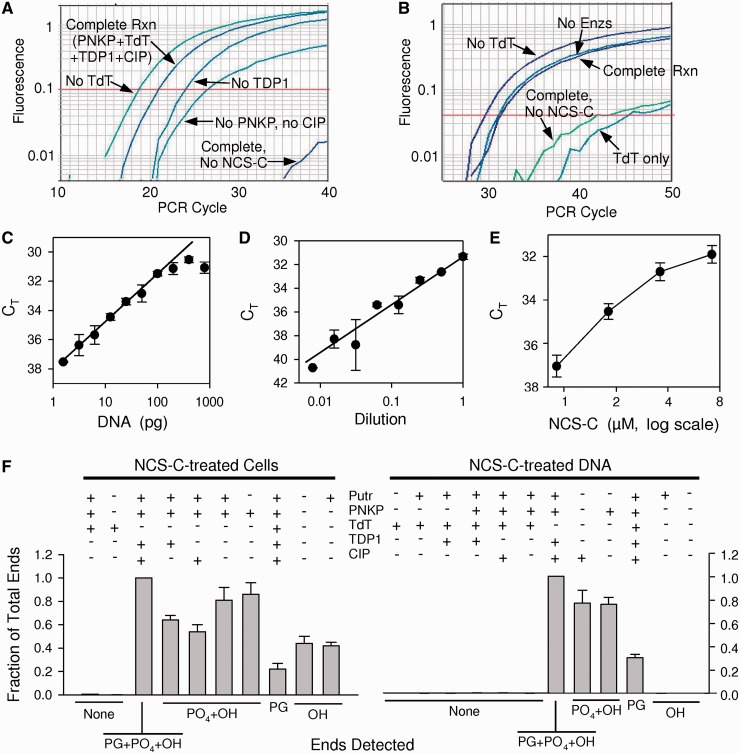
Detection and quantitation of 3′-PG, 3′-phosphate and 3′-hydroxyl DSB termini on NCS-C-induced DSBs. (**A**) Detection of DSBs in a cloned Alu sequence. A plasmid harboring the BLUR8 Alu sequence was treated with 100-nM NCS-C, then with NaBH_4_ and putrescine and finally with PNKP, TdT, TDP1 and CIP. TdT, TDP1 or NCS-C was omitted where indicated. DSB ends were then ligated to an anchor and quantitated by Taqman PCR. (**B**) Detection of DSB ends in DNA from cells treated with 5 -μM NCS-C. Chemical and enzymatic treatments and Taqman PCR, were the same as in (A), except that only 50 pg of ligated template DNA was added and a consensus Alu PCR primer was used. Various treatments were omitted as indicated. (**C**) Titration of the PCR assay for 3′-PG DSBs. Conditions are the same as for the Complete Rxn in (B), except that various dilutions of the ligated DNA were used as PCR template. The threshold cycle C_T_ is plotted, (threshold level of 0.04 fluorescence units is indicated by red line in B). The slope of the best-fit line was exactly one PCR cycle per 2-fold dilution. (**D**) Same as (C), except that to mimic decreasing levels of 3′-PG DSBs in DNA from treated cells, the same ligated DNA was diluted in ligated DNA from untreated cells, keeping total template DNA constant at 50 pg. The best-fit slope was 1.28 PCR cycles per 2-fold dilution. (**E**) Dose response for formation of 3′-PG DSBs in SCAN1 lymphoblastoid cells by NCS-C. Cells were treated with NCS-C for 10 min at 37°C. (**F**) Detection and quantitation of 3′-PG, 3′-phosphate and 3′-hydroxyl DSB termini in DNA from NCS-C-treated cells (left), or in NCS-C-treated DNA (right). DNA was subjected to chemical and enzymatic manipulations as is in (B), with various treatments omitted as indicated. Ligatable DNA ends were then quantitated by LMPCR, and normalized to the sample where TdT treatment was omitted so that all 3′-PG, 3′-phosphate and 3′-hydroxyl DSB termini would be rendered ligatable. The types of ends that should be detected by each combination of treatments are listed below the graph. For simplicity, a change of one PCR cycle in C_T_ was assumed to correspond to a factor of 2 in the number of ligatable ends. C_T_ values from which these data were derived are shown in Supplementary Figure S3. In all panels, error bars show mean ± SEM for at least three PCR reactions.

To assess the ability of the assay to detect NCS-C-induced 3′-PG DSBs, a plasmid containing the cloned BLUR8 Alu sequence (16) was treated with NCS-C in the presence of glutathione, then subjected to the end-modification reactions shown in Figure 1A, followed by ligation-mediated Taqman PCR (LMPCR) using a primer corresponding to the specific Alu variant in BLUR8 (Figure 2A). Consistently, a robust amplification signal was generated, reaching threshold at cycle C_T_∼20. Gel electrophoresis of the PCR products revealed a prominent peak at the expected length of 65 bp, and only from the NCS-C-treated samples (Supplementary Figure S2). Omission of NCS-C, CIP, the anchor, DNA ligase or either of the primers completely eliminated any PCR signal, even after 40 cycles. When TdT was omitted, so that both 3′-PG-terminated and 3′-phosphate-terminated DSBs would be ligated and amplified, there was a decrease in C_T_ of approximately two cycles, consistent with biochemical studies showing that NCS-C induces more 3′-phosphate than 3′-PG DSBs at AGT•ACT sites (24,25). Importantly, omitting TDP1, so that all 3′-PG termini would remain intact, increased C_T_ by about three cycles, corresponding to an 8-fold decrease in measured DSBs. Thus, the assay apparently can detect NCS-C-induced 3′-PG-terminated DSBs, and alterations in the procedure yield changes in signal intensity that would be predicted by the model (Figure 1A).

### Detection of DSBs induced by NCS-C in cells

To determine whether the assay could detect damage in live cells, TDP1-deficient lymphoblastoid cells (12) were suspended in cold PBS, and treated with 5-μM NCS-C for 10 min at 37°C. DNA was rapidly isolated using Qiagen DNeasy columns and subjected to the same chemical and enzymatic treatments, followed by SYBR-PCR-based determination of DNA concentration (Supplementary Figure S1), and LMPCR, replacing the BLUR8 primer with a primer corresponding to bp 186–205 of the human consensus Alu sequence (28). For DNA from cells treated with NCS-C for 10 min, a robust real-time PCR signal was consistently generated, with C_T_∼32–33 (Figure 2B). Surprisingly, the real-time signal reached a plateau at ∼100 pg of template DNA (Figure 2C), with higher concentrations of template producing a flattening of the amplification curve (data not shown). This effect, which was not seen with the cloned BLUR8 sequence, may have been due to an accumulation of DNA from one-sided amplification of the large number of Alu repeats in genomic DNA. Subsequent experiments were therefore performed with 50 pg of template. Below 50–100 pg, C_T_ increased by exactly one cycle for each 2-fold dilution of the DNA from treated cells as expected (Figure 2C).

As further verification of the assay, DNA from the treated cells was subjected to various combinations of enzyme treatments designed to detect specific types of DSB termini. C_T_ values for the resulting LMPCR are shown in Supplementary Figure S3, whereas Figure 2F shows the relative abundance of various types of DNA ends calculated from these data. The values are normalized to that obtained for treatment with only TDP1 and CIP, which should reflect the total of 3′-PG, 3′-phosphate and 3′-hydroxyl DSB ends. Just as with the NCS-C-treated plasmid, omission of TdT increased the signal, whereas omission of TDP1 and CIP dramatically decreased it as predicted. DNA that was not treated with any enzymes gave a signal comparable with that of the full complement of enzymes, indicating that many DSBs with 3′-hydroxyl termini were present in the cellular DNA, probably reflecting rapid dephosphorylation of 3′-phosphate DSBs by PNKP. To test this interpretation, the assay was applied to isolated human genomic DNA that was treated directly with NCS-C at a concentration (100 nM) that gave a real-time signal similar to that of DNA from treated cells. In this case, samples not subjected to any enzymatic processing yielded no signal at all, as did samples subjected to any of several enzyme treatments that would not generate 3′-hydroxyl termini from NCS-C-induced breaks. Otherwise, the results for NCS-C-treated DNA were similar to those for DNA from treated cells, with omission of TdT again increasing the signal as predicted. From the decrease in C_T_ that resulted from omission of TdT, the fraction of DSBs with PG termini was estimated to be 25% for treated cells and 33% for treated DNA. These values are comparable with those estimated from direct electrophoretic analysis of NCS-C-induced DSBs in end-labeled oligomers (25).

To assess whether the Taqman PCR signal was a quantitative measure of 3′-PG DSB ends in cells, DNA from cells treated with NCS-C was serially diluted in DNA from vehicle-treated cells (Figure 2D). The signal decreased as expected with each dilution, and a logarithmic plot of C_T_ versus dilution factor was approximately linear. Each 2-fold dilution in undamaged DNA increased C_T_ by ∼1.28 cycles, although there was considerable variation in C_T_ for extremely dilute samples. Thus, dilution in undamaged DNA yielded a greater-than-expected increase in C_T_. This result, as well as the shallow slopes of the amplification curves for samples containing low levels of 3′-PG DSBs, suggest that amplification may become slightly less efficient at late cycles, perhaps because of a buildup of amplified Alu DNA in the reactions. Similarly, when cells were treated with various concentrations of NCS-C, the real-time signal increased with NCS-C concentration (Figure 2E).

High-throughput sequencing of the PCR products formed in reactions to detect 3′-PG DSBs confirmed that NCS-treated cells or DNA yielded ∼20 times more products consistent with ligation of the anchor to the NCS-C DSB hotspot than did untreated controls (Supplementary Table S2). Moreover, for products in the appropriate size range, the consensus of sequences linked to the anchor matched the Alu consensus sequence immediately 5′ to this DSB hotspot, but only for NCS-C-treated samples (Supplementary Figures S4 and S5), strongly implying that the observed Taqman signals derive from these DSBs. Unexpectedly, the longer 5′-tailed primers required for high-throughput sequencing yielded more robust Taqman signals than the original primers, and so were used for most subsequent experiments. Overall, these results show that the assay is capable of quantifying PG-terminated NCS-C-induced DSBs with reasonable accuracy, and that by appropriate modifications, DSBs bearing 3′-phosphate and 3′-hydroxyl termini can be measured as well.

### DSB end processing in lymphoblastoid cells

**Figure 3.**
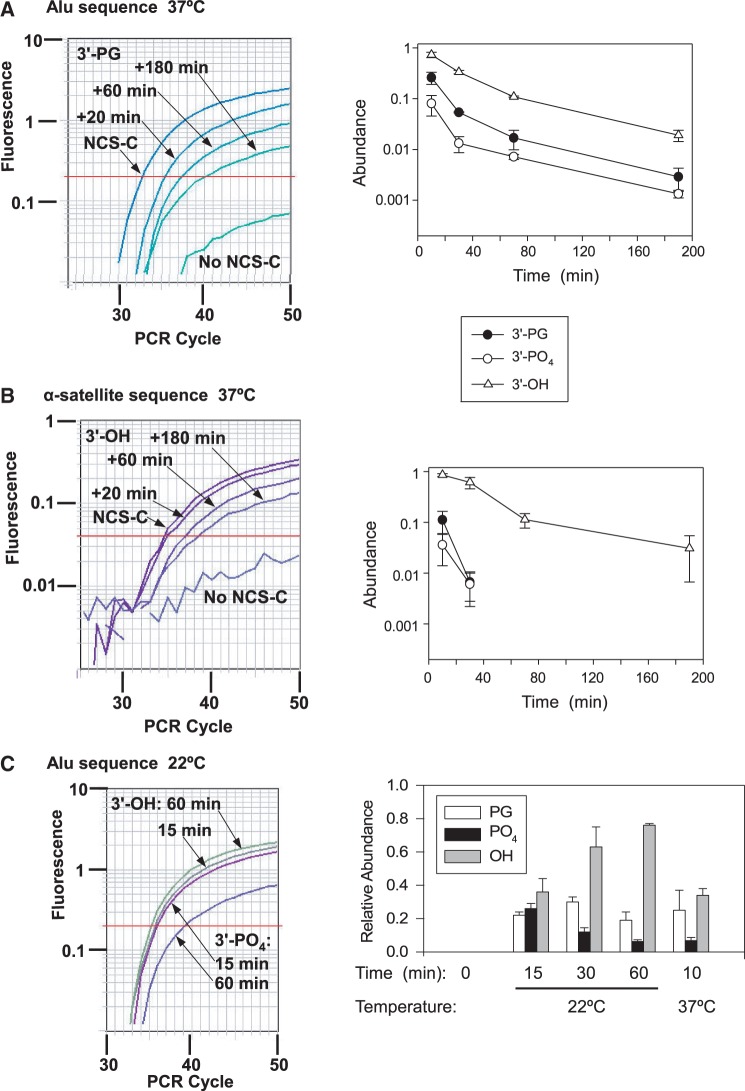
Kinetics of 3′ processing of DSBs in lymphoblastoid cells. (**A**) Cells in PBS were treated with 5 -μM NCS-C for 5 min at 0°C, and then for the indicated times at 37°C, with complete medium added after 10 min. DNA was isolated and subjected to the complete enzyme reactions for detection of 3′-PGs, TdT and CIP only for detection of 3′-phosphates or no enzymes for detection of 3′-hydroxyls (OH), then subjected to LMPCR. Typical amplification profiles for 3′-PG lesions are shown. Values (mean ± SEM from three independent experiments, each with three to four PCR reactions per sample) are normalized to the total of all three lesions at 10 min. (**B)** DNA samples from the experiment shown in (A) were subjected to LMPCR with primers and a probe specific to α-satellite DNA, and similarly analyzed. Owing to the weaker LMPCR signal for the satellite sequence, PG and PO_4_ data at >30 min were indistinguishable from background. Profiles for 3′-hydroxyl lesions are shown. (**C**) Cells were incubated in PBS at 0°C for 5 min and then at 22°C for the indicated times or for 10 min at 37°C, and the same three types of DSB ends were determined. The abundance of each type of lesion, calculated from C_T_ values, was normalized to the average level of total lesions at 30 and 60 min in each experiment. Error bars show mean ± SEM from four experiments.

To assess the abundance of particular DSB end structures as a function of time after treatment, immortalized lymphoblastoid cells were treated with NCS-C, and the DNA was harvested after various periods of repair. Levels of 3′-PG, 3′-phosphate and 3′-hydroxyl DSB ends were then assessed as above (Figure 3A).

All lesions reached a maximum within the 10-min treatment period, as expected from rapid activation and degradation of NCS-C. Moreover, all lesions decreased at least 8-fold within 1 h, indicating relatively rapid processing. However, even though NCS-C induces about twice as many 3′-phosphate as 3′-PG DSBs at AGT•ACT sites (25), 3′-PG-terminated DSBs in cellular DNA outnumbered 3′-phosphate DSBs by at least a factor of ∼3 at early time points, suggesting that the 3′-PGs were less efficiently processed. To test this inference, cells were treated with NCS-C at 22°C for up to 1 h (Figure 3C). At this temperature, measured 3′-phosphate DSBs peaked at 15 min, when their incidence was comparable with that of 3′-PG and 3′-hydroxyl DSBs, but then declined to <10% of all lesions by 1 h, while 3′-hydroxyl DSBs accumulated. These results suggest that 3′-phosphates (as well as abasic sites, which would be detected as 3′-phosphates in the assay) on DSB ends are removed rapidly in cells, presumably by PNKP (3′-phosphates) and APE1 (abasic sites). Interpretation of the results is complicated by the several processes that are occurring simultaneously: the formation of the three types of lesions by NCS-C, the enzymatic conversion of 3′-PGs to 3′-phosphates, and of 3′-phosphates (as well as abasic sites) to 3′-hydroxyls, and then the further processing and eventual religation of the 3′-hydroxyl DSBs. Nevertheless, the results are most consistent with a model where, at 22°C, most of the initial damage is complete within 15 min and most of the initial 3′-phosphate ends are converted to 3′-hydroxyls within 30 min, whereas there is relatively little processing of 3′-PG and 3′-hydroxyl ends at this temperature. The distribution of lesions after 60 min at 22°C is similar to that seen after 10 min at 37°C, except that there were fewer 3′-hydroxyl lesions remaining at 37°C, suggesting that further processing of the 3′-hydroxyls was more temperature-dependent than their formation from 3′-phosphates.

PNKP is regarded as the enzyme primarily responsible for dephosphorylation of 3′-phosphate DNA termini (as well as phosphorylation of 5′-hydroxyl termini), and the radiosensitivity of PNKP-knockdown cells (32) presumably reflects loss of this function. The imidopiperidine A12B4C3 reversibly inhibits the phosphatase activity of PNKP non-competitively by disrupting PNKP structure (33). Surprisingly, A12B4C3 had no discernable effect on the formation or removal of 3′-phosphate termini (Supplementary Figure S6). PNKP knockdown by shRNA in A549 cells also had little, if any, effect on 3′-phosphate kinetics as measured by the Taqman assay (Supplementary Figure S7), raising the possibility that some alternative factor can substitute for PNKP in dephosphorylating 3′-ends of DSBs.

To examine repair in heterochromatin, the Taqman PCR assays were repeated using primers and probe specific to an AGT•ACT site in the highly repeated α-satellite DNA sequence (21), which should be predominantly heterochromatic. Because of an intrinsically weaker signal for this site, accurate kinetic repair data could only be obtained for 3′-hydroxyl DSB termini, and these were apparently processed at a rate similar to those in the (predominantly euchromatic) Alu sequences (Figure 3B). Although 3′-PG- and 3′-phosphate-terminated DSBs could only be detected at the earliest time points, they nevertheless appeared to be eliminated about as quickly as those in Alu sequences. (As predicted, the α-satellite probe yielded no signal under any condition with the Alu-specific primer, and vice versa; data not shown.)

### Defective processing of 3′-PG DBSs in TDP1-deficient SCAN1 cells

**Figure 4.**
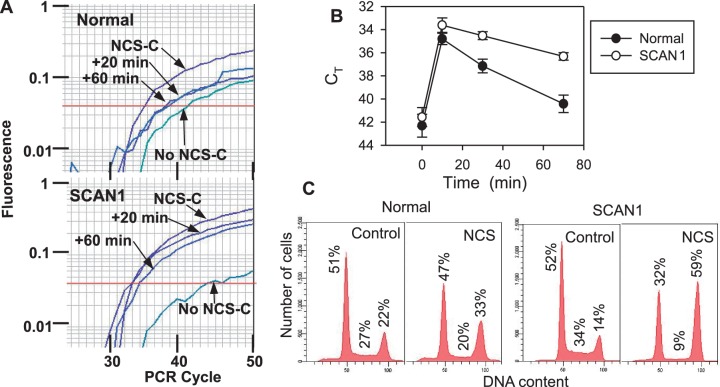
Increased persistence of 3′-PG DSB ends in SCAN1 cells. (**A**) Cells were treated with 5 -μM NCS-C for 10 min at 37°C, and either harvested immediately or after incubation in complete medium for 20 or 60 min, and then 3′-PG DSB termini were detected by Taqman PCR. (**B**) Pooled real-time data (C_T_ values, mean ± SEM) from four separate experiments, two with cells from patients 1646 (normal) and 1662 (SCAN1) and two with cells from patients 1668 (normal) and 1664 (SCAN1), with three to four PCR reactions for each sample. (**C**) Cell cycle profiles of normal (1646) and SCAN1 (1662) cells before and after treatment with 1-nM neocarzinostatin (NCS) for 1 h. G_1_, S and G_2_ fractions are indicated. Similar results were seen at other time points and in replicate experiments (Supplementary Figure S8).

In whole-cell extracts, essentially all processing of protruding 3′-PG DSB termini appears to be attributable to TDP1 (34). To assess a role for TDP1 in PG processing in intact cells, TDP1-mutant lymphoblastoid cells from SCAN1 patients, or normal cells from unaffected relatives, were treated with NCS-C, and incubated for 20 or 60 min to allow repair (Figure 4A). As described above, 3′-PG DSBs were readily detected by LMPCR, and these lesions decreased with increasing repair time in all cell lines. However, pooled data from four independent experiments with two different sets of normal and SCAN1 cell lines (Figure 4B) showed that although the initial level of 3′-PG DSBs following the 10-min treatment was similar in all cell lines, the lesions were eliminated two to three times faster in normal cells than in SCAN1 cells (*P* < 0.001 by Student’s *t*-test at both 20 and 60 min). Moreover, SCAN1 cells showed a more severe and more persistent G_2_ arrest than normal cells following NCS treatment, consistent with a deficiency in repair of NCS-induced DSBs (Figure 4C and Supplementaary Figure S8). Thus, these results suggest a major role for TDP1 in removing 3′-PG termini from DSB ends, although it appears these lesions can be slowly processed by other enzymes when TDP1 is absent. It should be noted that the assay does not distinguish the nature of the end processing; for example, resection of the 5′ terminus without any 3′-PG processing would be indistinguishable from alteration of the 3′-PG terminus, as either event would preclude ligation of the lesion to the anchor and result in loss of the PCR signal. In other words, the assay gives a measure of the ends that remain completely intact and unprocessed.

Processing of 3′-PG termini was also examined in TDP1-knockdown HCT116 colon carcinoma cells. However, an apparent decrease in nuclear TDP1 by 75 ± 8% did not confer a discernable defect in 3′-PG processing (Supplementary Figure S9).

### Increased ratio of 3′-hydroxyl to 3′-PG DSB ends in DNA-PK-deficient cells

**Figure 5.**
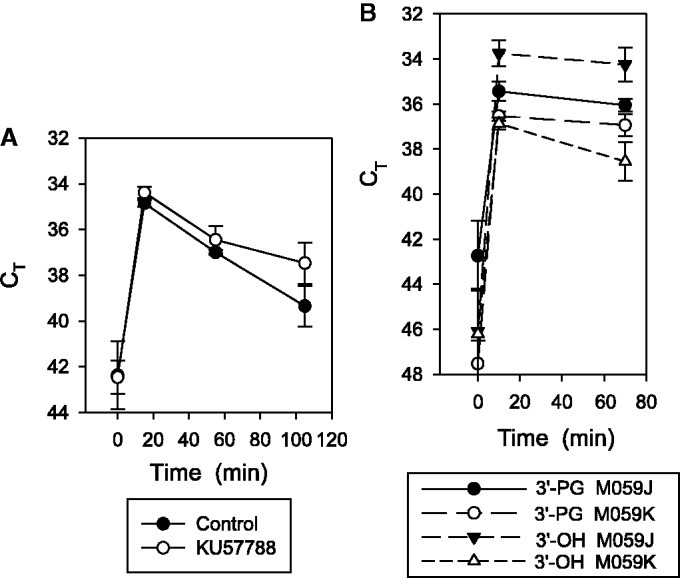
Effect of DNA-PK inhibition or absence of DNA-PK on 3′-PG processing. (**A**) Normal lymphoblastoid cells (patient 1646) were grown in the presence 10 -μM KU-57788 (or 0.4% DMSO) for 2 h, treated with NCS-C as in Figure 4 and then diluted into complete medium with or without KU-57788 and harvested at the indicated times for determination of 3′-PG DSBs. (**B**) M059K or DNA-PK-deficient M059J glioma cells were trypsinized, treated with NCS-C as in (A), then harvested either immediately or after incubation in complete medium for 1 h. Levels of 3′-PG and 3′-hydroxyl DSBs were then determined by LMPCR. Error bars show mean ± SEM for four experiments. Differences between M059J and M059K in the ratio of PG- to hydroxyl-terminated DSB ends were significant (*P* < 0.01) at both time points.

DNA-PK binds to DSB ends through its Ku subunits and regulates nucleolytic DNA end processing during V(D)J recombination, primarily through trans-autophosphorylation at several clusters of SQ/TQ sites in its catalytic subunit (35). KU-57788 is an aryl-substituted chromenone that binds competitively to the ATP binding pocket of DNA-PK and thereby reversibly inhibits its kinase activity, but does not affect the closely related ATM or ATR kinases (36). To determine whether DNA-PK also regulates resolution of chemically damaged ends, 3′-PG DSB termini were measured in NCS-C-treated normal lymphoblastoid cells in the presence or absence of KU-57788 (Figure 5A), at a concentration previously reported to effectively block DNA-PK autophosphorylation in cells (37). KU-57788 was similarly effective in inhibiting DNA-PK in lymphoblastoid cells, as verified by a 10-fold reduction in DNA-PK autophosphorylation at S2056 (Supplementary Figure S10). There was a slight inhibitory effect of DMSO solvent alone on 3′-PG processing, and in some experiments inhibition of DNA-PK by KU-57788 appeared to decrease the rate of 3′-PG processing, but this decrease was not seen consistently and was not statistically significant (Figure 5A). A similar lack of effect on 3′-PG processing was seen in A549 cells, even though KU-57788 markedly increased the persistence of γH2AX foci, thus confirming its inhibitory effect on repair overall (Supplementary Figure S11).

M059J and M059K cells are a matched pair of glioma lines that originated from same tumor, but M059J cells are completely deficient in DNA-PK, while M059K cells have normal DNA-PK expression (14). To examine the possible effects of a lack of DNA-PK on 3′-PG processing, 3′-PG and 3′-hydroxyl DSB ends were compared in these two cell lines following treatment with NCS-C (Figure 5B). A difference in the relative proportions of PG- and hydroxyl-terminated DSB ends was apparent within the first 10 min of treatment, at which time DNA-PK-deficient M059J cells consistently had more hydroxyl than PG ends (by a factor of 4.0 ± 0.9, N = 4), while M059K cells always had more PG than hydroxyl ends (by a factor of 1.46 ± 0.23). Both PG- and hydroxyl-terminated ends were eliminated more slowly in these glioma cell lines than in the lymphoblastoid cells, but the difference between M059J and M059K cells in the ratio of 3′-PG to 3′-hydroxyl ends still persisted 60 min after treatment, and was significant (*P* < 0.01) at both time points. Attempts to obtain reproducible data at shorter time points were unsuccessful. Overall, the results suggest that, at early times in the repair process, conversion of PG- to hydroxyl-terminated DSB ends is faster when access to the ends is not regulated by DNA-PK.

## DISCUSSION

LMPCR can detect, with great sensitivity, any lesion that can be converted to a ligatable strand break. This technique has thus been used extensively to measure site-specific formation and repair of a variety of DNA base lesions (38) as well as the production of DSBs by ectopic rare-cutting endonucleases (39). However, use of LMPCR for studying repair of free radical-mediated DSBs is particularly challenging because of the extreme toxicity of these lesions and the lack of agents that can produce them in a site-specific and time-specific manner. In the present work, these difficulties have been overcome by taking advantage of the rapid uptake and activation/inactivation of NCS-C, the modest sequence preferences and defined structure of NCS-C-induced DSBs, the high copy number of the human Alu repeat and the exquisite sensitivity and selectivity of Taqman PCR. A variety of reconstruction and control experiments indicate that the resulting Taqman method, and variations thereof, can detect and quantify 3′-PG, 3′-phosphate and 3′-hydroxyl DSBs formed by NCS-C, both in cells and in isolated DNA. This is to our knowledge the first successful attempt to specifically examine the processing of free radical-induced DSB termini in cells.

Analysis of DSB termini at various times after NCS-C treatment of lymphoblastoid cells indicates that intact 3′-phosphate DSBs disappear rapidly, largely within the initial 10-min treatment period at 37°C, and within 30 min even at 22°C. This processing likely reflects the action of PNKP, which is recruited to DSBs by XRCC4 (40), and whose phosphatase activity is highly specific for 3′ DNA ends (41). However, the surprising lack of effect of PNKP knockdown and inhibition raises the possibility of an alternative mode of phosphate removal. DSBs with 3′-PG termini, on the other hand, are more persistent, remaining intact for 1 h at 22°C, but being mostly eliminated within 30 min at 37°C. This is consistent with studies in cell extracts, which show partial TDP1-dependent conversion of 3′-PG to 3′-hydroxyl DSBs without detectable 3′-phosphate intermediates (34), implying that the 3′-phosphates formed by TDP1 were rapidly removed. Thus, as expected, hydroxyl-terminated DSBs, which are not induced directly by NCS-C, accumulate quickly in cells (presumably from hydrolysis of 3′-phosphate DSBs) and then disappear with kinetics similar to those of 3′-PG DSBs (Figure 3A). At later times, loss of the remaining DSBs, regardless of terminal structure, proceeds more slowly. While this could reflect slower repair of DSBs in less accessible chromatin regions, there was no dramatic difference in these kinetics in satellite DNA, which should be predominantly heterochromatic, compared with the broadly distributed Alu repeats. Thus, while other studies indicate that DSB rejoining in heterochromatin requires several hours, much slower than in euchromatin (42), the present results suggest that the initial end-processing steps occur much more quickly, regardless of chromatin context.

In TDP1-mutant SCAN1 cells, the rate of 3′-PG processing was 2–3-fold slower than in normal cells, suggesting that most, but not all, of the 3′-PG DSB processing is attributable to TDP1. Overall, the PG terminus appears to be somewhat of an orphan lesion. While TDP1 and APE1 are each capable of resolving 3′-PG DSB termini (APE1 on blunt and recessed ends only), both enzymes act on PG termini much less efficiently than on their canonical substrates, i.e. 3′-phosphotyrosyl termini and abasic sites, respectively (5,6). Artemis endonuclease can also resolve 3′-PG termini, by excising a 3′-PG mono- or oligonucleotide, but this reaction is also slow, particularly for short 3′ overhangs and blunt ends (7,43).

In whole-cell or nuclear extracts of TDP1-mutant SCAN1 lymphoblastoid cells, protruding 3′-PG DSB termini remain intact and unprocessed for many hours, while in normal cell extracts most are converted to 3′-hydroxyls within 30 min (34). Thus, the deficit in PG processing seen in SCAN1 extracts is significantly greater than that seen in cells, suggesting additional backup repair pathways, not fully expressed in extracts, are active *in vivo*. We showed previously that, if presented with a partially complementary DSB with one 3′-PG and one 3′-hydroxyl terminus, the mammalian non-homologous end-joining system is capable of patching and ligating the 3′-hydroxyl strand, even while the closely opposed 3′-PG break in the opposite strand remains unrepaired and retains its 3′-PG terminus (44). This remaining break could then presumably be repaired as a single-strand break by APE1, XRCC1, DNA polymerase beta and DNA ligase III. This alternative repair pathway may explain the residual processing of 3′-PG DSBs in SCAN1. However, such a pathway would require some nuclease or other mechanism to remove the 5′-aldehyde terminus and expose a 5′-phosphate to allow ligation of the opposite strand of NCS-C-induced DSBs. In any case, the severe G2 arrest seen in NCS-treated SCAN1 cells (Figure 4C) suggests that lack of TDP1-mediated 3′-PG processing may be biologically significant. The reason for the apparent lack of a 3′-PG processing defect in TDP1-knockdown cells is presently unclear. As in any shRNA experiment, it is difficult to exclude the possibility that the residual ∼25% of TDP1 activity was sufficient for biological function.

DSBs with protruding 3′-PG termini in both strands would of course strictly require a DSB-specific mechanism of PG resolution, but such lesions are not produced by radiomimetic natural products (3,4) and may be rare among radiation-induced DSBs. Whether deficiency in 3′-PG removal contributes to ataxia, cerebellar degeneration or other symptoms of SCAN1 is unknown. Ostensibly, the deficiency in processing TDP1’s canonical substrate, i.e. 3′-tyrosyl-linked peptides (45) derived from topoisomerase I reaction intermediates, would appear more likely. However, Tdp1-deficient *Saccharomyces cerevisiae* show spontaneous death in stationary phase that is independent of topoisomerase I (46), suggesting that in this organism, toxic lesions other than 3′-tyrosyl-linked peptides accumulate when Tdp1 is absent.

DNA-PK is thought to regulate DSB end processing during repair, primarily through autophosphorylation in *trans* (47). DNA-PK inhibitors such as KU-57788/NU-7441 severely inhibit DSB repair in cells (48), and studies of V(D)J recombination at engineered loci, as well as *in vitro* experiments with purified proteins, suggest that autophosphorylation is required to make DSBs accessible to end-processing enzymes (49,50). Thus, the finding that KU-57788 has little, if any, effect on resolution of 3′-PG DSB ends is surprising, and suggests that when DNA-PK autophosphorylation stalls, there are mechanisms in place to ensure that blocked DSB ends are still resolved, even if the ultimate rejoining of the breaks is slower and/or less efficient. On the other hand, absence of DNA-PK, as in M059J cells, appears to significantly accelerate PG removal, consistent with a role of DNA-PK in regulating processing. However, strikingly, the 3′-hydroxyl ends thus generated appear to be as stable in M059J as in M059K cells, suggesting that DSB ends are still protected from inappropriate nucleolytic degradation even when not sequestered by DNA-PK.

While originally designed for the express purpose of quantitating 3′-PG DSB termini, the methods described above can in principle be modified to detect other putative intermediate steps in repair, including nucleolytic processing of DNA ends after removal of terminal blocks. Although rare-cutting nucleases have been extremely helpful for such studies, their activity is difficult to turn off once induced. In contrast, NCS-C acts quickly and then degrades, making it a more suitable damaging agent for examining the time course of early events in repair. The caveat is that, to be detected by this method, any such putative repair intermediate would have to be frequent enough and persistent enough to constitute, at the time of assay, a substantial fraction of the initial lesions induced.

## SUPPLEMENTARY DATA

Supplementary Data are available at NAR Online.

## FUNDING

National Cancer Institute CA40615 from US Public Health Service. Funding for open access charge: NIH CA40615.

*Conflict of interest statement*. None declared.

## Supplementary Material

Supplementary Data
