# Supplementary material for: Tracking the processing of damaged DNA double-strand break ends by ligation-mediated PCR: increased persistence of 3′-phosphoglycolate termini in SCAN1 cells
Source: Nucleic Acids Res. 2013 Dec 25;42(5):3125–37. doi: 10.1093/nar/gkt1347 (PMC3950721; doi:10.1093/nar/gkt1347)
Supplement: Supplementary Data [file supp_42_5_3125__index.html]

Tracking the processing of damaged DNA double-strand break ends by ligation-mediated PCR: increased persistence of 3′-phosphoglycolate termini in SCAN1 cells — Supplementary Data 

# Tracking the processing of damaged DNA double-strand break ends by ligation-mediated PCR: increased persistence of 3′-phosphoglycolate termini in SCAN1 cells

## Supplementary Data

files

**Files in this Data Supplement:**

- Supplementary Data - pdf file
