# Supplementary material for: Tracking the processing of damaged DNA double-strand break ends by ligation-mediated PCR: increased persistence of 3′-phosphoglycolate termini in SCAN1 cells
Source: Nucleic Acids Res. 2013 Dec 25;42(5):3125–37. doi: 10.1093/nar/gkt1347 (PMC3950721; doi:10.1093/nar/gkt1347)
Supplement: Supplementary Data [file supp_gkt1347_nar-01292-d-2013-File007.pdf]

Supplemental Table I. Primers used for LaRoche 454 sequencing

Forward:	454 adaptor	Key	Barcode	Alu (Fwd) or Anchor (Rev)
Untreated cells*	CCATCTCATCCCTGCGTGTCTCCGAC	TCAG	TATCTGATAG	GGCAGGAGTATCGCTTGAAC
Treated cells	CCATCTCATCCCTGCGTGTCTCCGAC	TCAG	TCGTGACATG	GGCAGGAGTATCGCTTGAAC
Untreated DNA	CCATCTCATCCCTGCGTGTCTCCGAC	TCAG	TCTGATCGAG	GGCAGGAGTATCGCTTGAAC
Treated DNA	CCATCTCATCCCTGCGTGTCTCCGAC	TCAG	TGACATCTCG	GGCAGGAGTATCGCTTGAAC
Reverse:	CCTATCCCCTGTGTGCCTTGGCAGTC	TCAG	———	TGCAACTCTGCGTCAAATCG

*The primer for untreated cells, along with the reverse primer, was routinely used for Taqman PCR assays as well.

Supplemental Table II. Summary of 454 sequencing of PCR products*

**Condition**	Number of Reads	Reads containing Taqman probe (%)	Average length between primers
Untreated DNA	16,384	89 (0.54%)	40.38
Untreated Cells	16,522	55 (0.33%)	28.39
NCS-C-treated DNA	23,133	2576 (11.1%)	32.51
NCS-C-treated Cells	26,588	3029 (11.4%)	31.37

*DNA treated with 100 nM NCS-C or DNA from cells treated with 5 μM NCS-C were subjected to the complete chemical stabilization and enzymatic reactions to detect 3′-PG DSB ends, followed by ligation-mediated real-time PCR using long primers containing 5′ tails to facilitate LaRoche 454 sequenging. For each condition, PCR product sequences between the primers were screened for presence of the Taqman probe corresponding to the predicted joint between NCS-C-induced DSBs and the anchor.

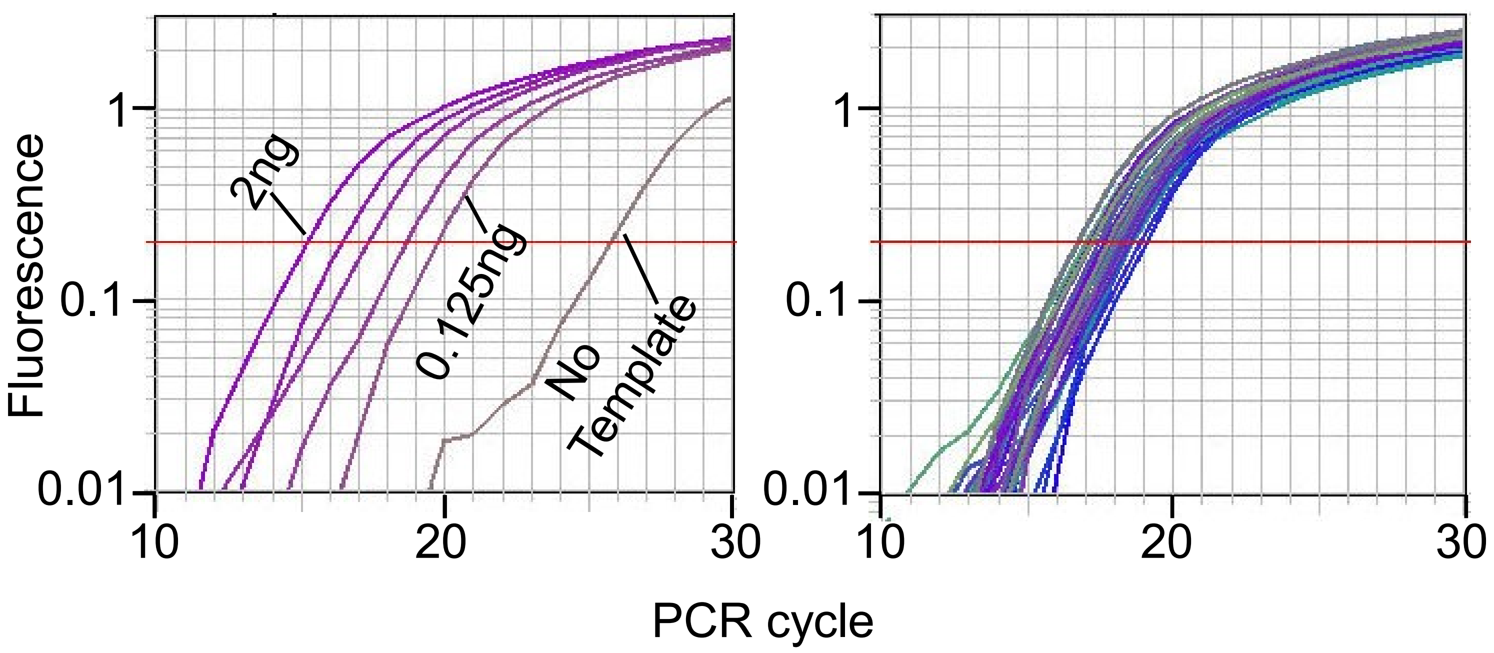

Supplemental Figure 1. Estimation of DNA concentration by Alu PCR. Left: To generate a standard concentration curve, DNA islolated from human 184B5 cells was serially diluted 2:1 and subjected to real-time PCR with SYBR green detection. Reactions (20 µL) were performed in duplicate and contained 1x SYBR Green PCR Master Mix (Applied Biosystems), 0.5 µL of the DNA sample, 0.4 µM of each Alu primer (forward GTCAGGAGATCGAGACCATCCC; reverse TCCTGCCTCAGCCTCCCAAG) and 10 µg/mL BSA. PCR was conducted in an Applied Biosystems 9700HT cycler with a thermal profile consisting of 10 min at 50ºC and 5 min at 95ºC, followed by 35 cycles of 15 sec at 95ºC, 30 sec at 68ºC and 30 sec at 72ºC. Right: Aliquots of genomic DNA samples isolated from NCS-C-treated cells in a typical experiment were similarly analyzed following enzyme treatments, just before ligation to the anchor. Typically, $1/40^{th}$ of each sample was used for each of 2 replicate PCR reactions. Samples in a single experiment normally varied by no more than a factor of 4 in concentration. An equal amount of DNA from each sample, usually 1 ng, was then used for each ligation reaction.

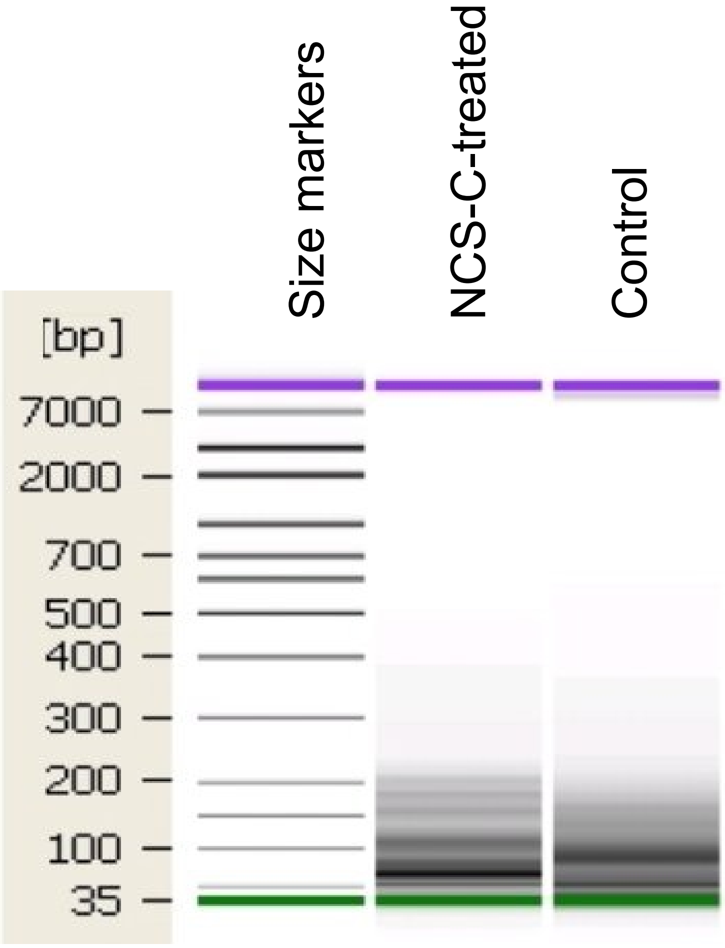

Supplemental Figure 2. Detection of NCS-C-induced DSBs at an AGT●ACT hotspot in a cloned Alu sequence. A plasmid containing the BLUR8 Alu sequence was treated with 100 nM NCS-C. The cleavage products were subjected to the complete chemical and enzymatic reactions for detection of 3′-PG DSB ends, and then ligated to an anchor and amplified by PCR. PCR products (1 μL) were subjected to microfluidic size analysis on an Agilent 2100 Bioanalyzer. The NCS-C-treated plasmid yielded a prominent band at 65 bp, as expected for ligation of the anchor to the NCS-C-induced DSB at the AGT●ACT hotspot at bp 224-226.

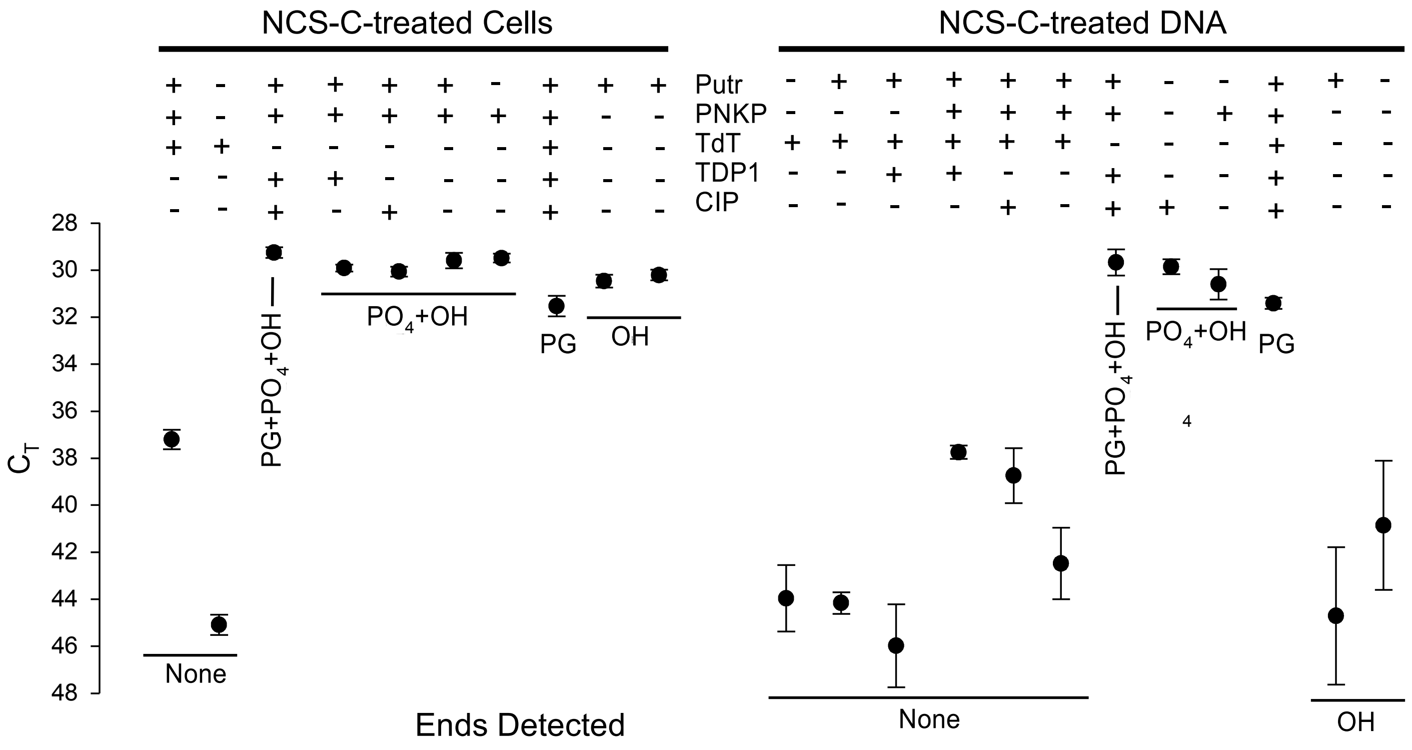

Supplemental Figure 3. Real-time PCR estimation of DSB termini for NCS-C-treated cells (left) or DNA (right). Following NCS-C treatment, the DNA was subjected to the indicated chemical and enzymatic manipulations (Putr = putrescine), and then ligatable ends were estimated by ligation-mediated real-time PCR. The types of termini expected to be detected for each combination of enzyme treatments are indicated below the data points. Error bars indicate mean ± SEM for at least three PCR reactions from a single treated sample. These data are replotted as a fraction of total lesions, in Fig. 2 of the Results.

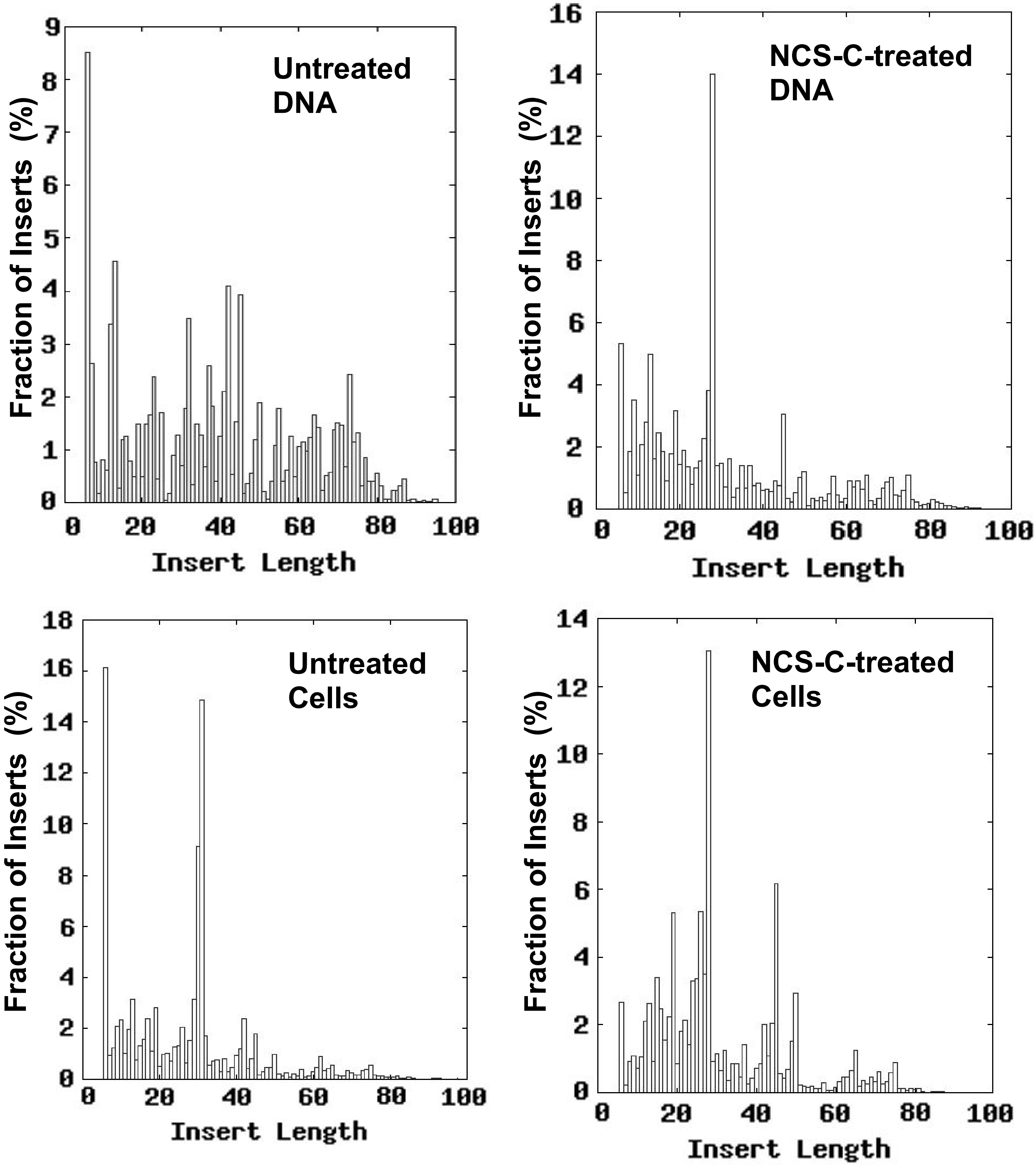

Supplemental Figure 4. Length distributions of inserts in products of LMPCR, determined by high-throughput LaRoche 454 sequencing. Products from treated DNA and cells show a distinct peak of 28-bp inserts, the expected length for ligation of the anchor to NCS-C-induced DSBs at the AG<u>T</u>●AC<u>T</u> hotspot at Alu bp 224-226. The inserts from untreated cells showed a peak of unknown origin at 31 bp. Methods: NCS-C-treated DNA or DNA from NCS-C-treated cells was subjected to the complete reaction for detection of 3′-PG DSBs. Ligation-mediated PCR reactions were performed using primers that were 5'-tailed with 454-specific priming sequences along with unique bar codes for each sample (Supplemental Table I), with the Taqman probe omitted. Three replicate 20 µl reactions for each sample were pooled and submitted to the Massey Cancer Center Nucleic Acids Shared Resource, where they were mixed with other uniquely bar-tagged samples and subjected to high-throughput sequencing on a LaRoche 454 instrument under standard conditions. The resulting sequence data were sorted according to the bar codes, and insert lengths (bp between ends of the primers) were determined.

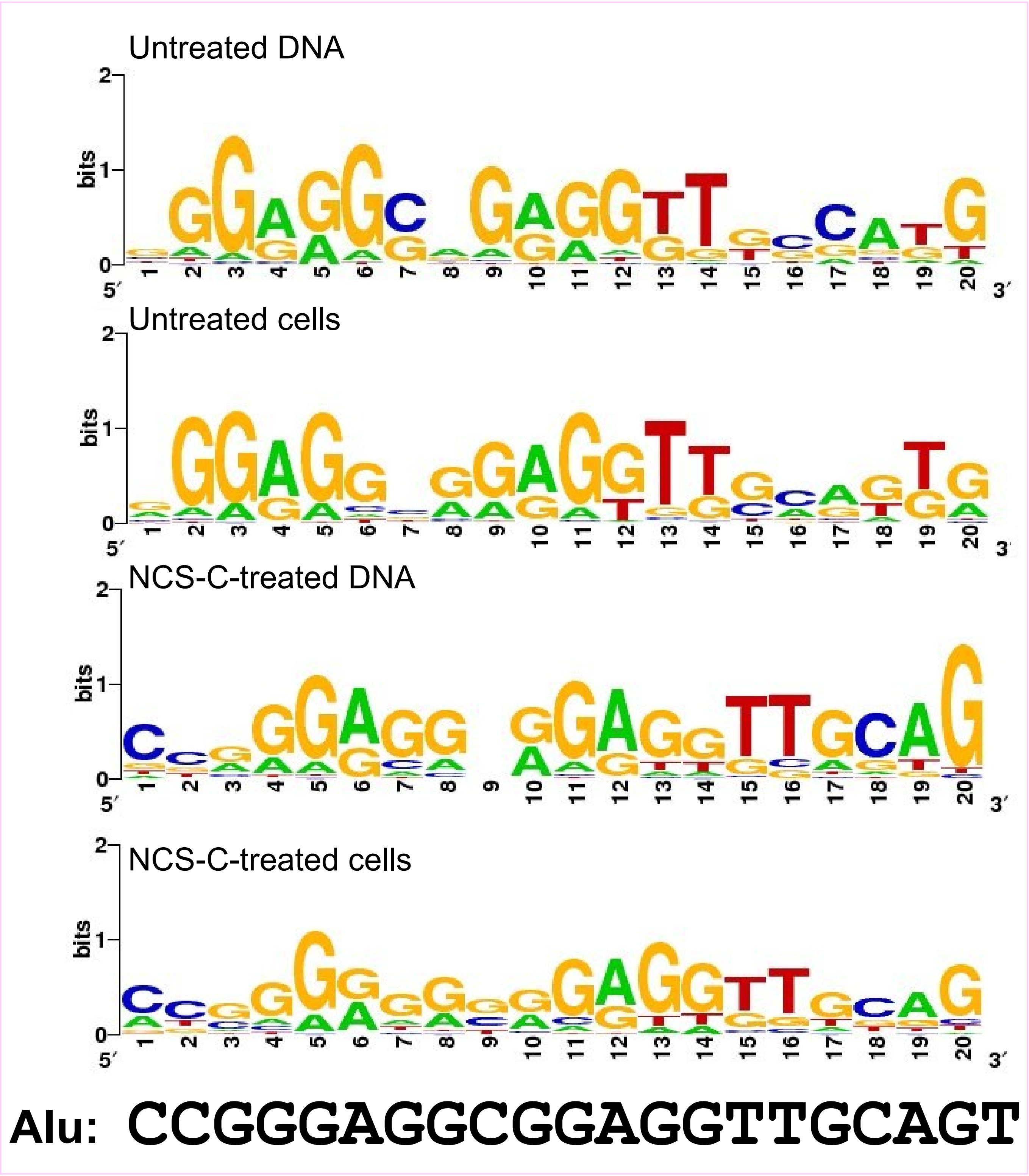

Supplemental Figure 5. Consensus sequences for the 20 bp 5′ to the anchor sequence in LMPCR products from NCS-C-treated or untreated cells and DNA. Each sequence logo shows the consensus sequence for all PCR products in the expected size range (26-30 bp between primers) for each condition. Overall height of each stack shows the degree of conservation at that position and heights of individual letters are in proportion to their frequency (Schneider & Stephens, 1990). NCS-C concentrations were 5 μM for cells and 100 nM for DNA, which gave approximately equal Taqman PCR signals; however, to avoid possible fluorescence interference, the Taqman probe was omitted from PCR reactions used for sequencing. The similarity to the Alu consensus confirms that NCS-C-induced DSBs at the bp 224-226 AGT hotspot in cellular DNA were indeed ligated to the anchor and amplified, and thus are presumably the basis of the observed Taqman signals. The consensus sequences for the control samples appear to derive from a DSB of unknown origin two bases 3′ to the NCS-C DSB hotspot.

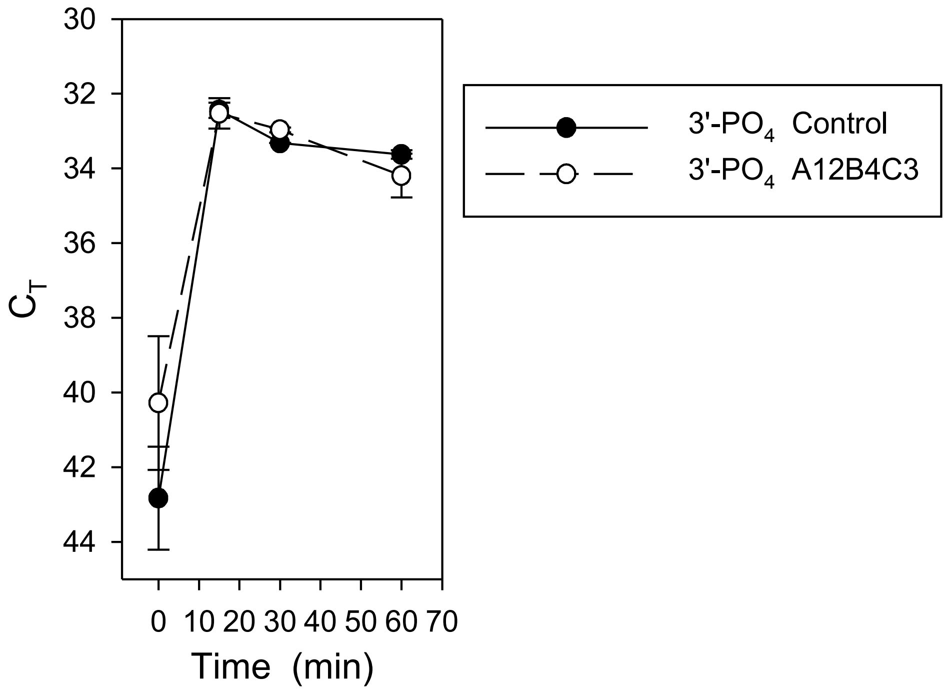

Supplemental Figure 6. Lack of effect of a PNKP inhibitor on formation and processing of 3′-phosphate DSBs. Normal lymphoblastoid cells (patient 1646 from Takashima et al. (2002)) were treated with 5 µM NCS-C in PBS in the presence or absence of the PNKP inhibitor A12B4C3 (Freschauf et al. 2009) and harvested after 15, 30 or 60 min at 22ºC. DNA was isolated from the cells and subjected to chemical stabilization, treatment with TdT plus CIP, and ligation-mediated Taqman PCR for detection of DSB ends bearing 3′-phosphate termini, as in Fig. 2. A12B4C3 (or 0.1% DMSO for control cells) was added to the cells 1 hr before NCS-C treatment and was present throughout the washes, resuspension and repair incubations. Data are from a single experiment and error bars show mean ± SD for two Taqman PCR reactions with each sample. Two additional independent experiments (not shown) showed a similar lack of inhibitor effect at either 22ºC or 37ºC. Although the data do not exclude the possibility that residual PNKP activity in the presence of inhibitor was sufficient for efficient 3′-phosphate removal, previous work in A549 cells showed that shRNA-mediated PNKP knockdown and treatment with A12B4C3 were epistatic in conferring radiosensitivity (Freschauf et al. 2009), suggesting that at least in those cells inhibition of the phosphatase activity of PNKP by A12B4C3 was essentially complete. However, we did not detect any enhancement of radiation-induced cell cycle arrest in A12B4C3-treated lymphoblastoid cells (as in Supplemental Fig. 8; data not shown).

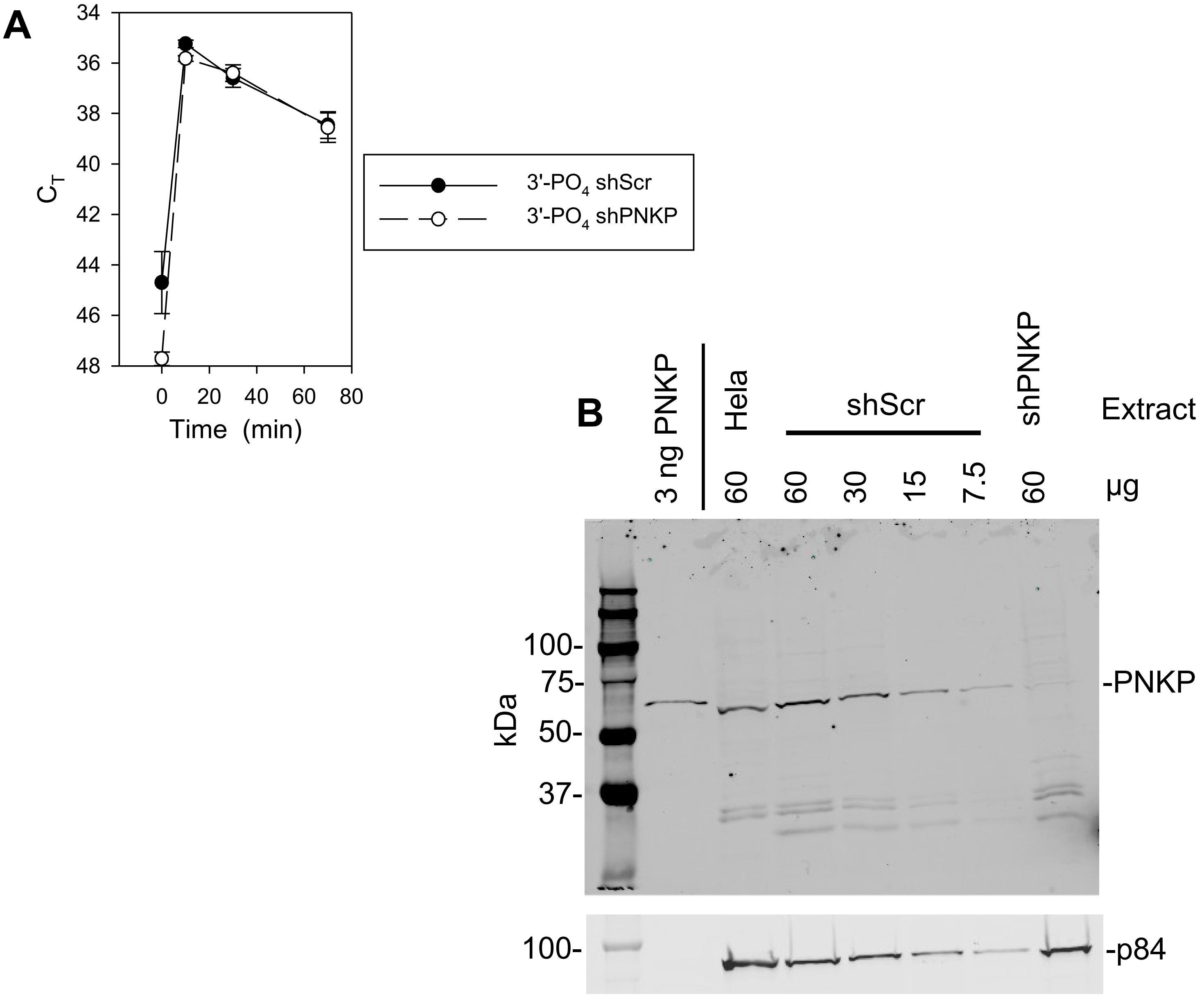

Supplemental Figure 7. Lack of effect of PNKP knockdown on formation and processing of 3′-phosphate DSB termini. (**A**) A549 lung carcinoma cells stably expressing either an shRNA targeting PNKP (shPNKP) (Rasouli-Nia et al. 2004), or a nontargeting scrambled shRNA (shScr), were trypsinized, washed and treated with 5 µM NCS-C in PBS for 10 min, then incubated in complete medium at 37ºC for 20 or 60 minutes to allow processing. DNA was isolated from the cells and subjected to chemical stabilization, enzymatic manipulations (TdT plus CIP) and ligation-mediated Taqman PCR for detection of DSB ends bearing 3'-phosphate termini, as in Fig. 2. Data are from a single experiment and error bars show mean ± SD for two Taqman PCR reactions with each sample. Two additional independent experiments (not shown) indicated a similar lack of effect of PNKP knockdown. (**B**) Verification of PNKP knockdown. Nuclear extracts were prepared from 5×10^5 shPNKP- or shScr-expressing A549 cells using a nuclear/cytosol fractionation kit (Biovision), and protein concentrations were determined using Pierce BCA reagent. Various quantities of the shScr extracts, or 60 µg of the shPNKP extract, were subjected to denaturing electrophoresis on 8% PAGE. The blot was first probed with an anti-PNKP mouse monoclonal antibody (1:1000 dilution, gift of Michael Weinfeld), followed by an Alexa 680 goat antimouse secondary antibody (Invitrogen). Following scanning on a LiCor blot reader, the blot was reprobed with mouse monoclonal antibody 5E10 against the p84 nuclear matrix protein (1:1000 dilution, GeneTex #GTX70220) as a loading control, followed by the same secondary antibody. Quantitation indicated a PNKP knockdown of approximately twelvefold. The blot was not stripped between probings, but the much weaker PNKP signal was negligible after probing for p84. PNKP knockdown cells and the PNKP antibody were provided by Michael Weinfeld, Cross Cancer Institute.

Supplemental Figure 8 (on next page). Increased sensitivity of SCAN1 cells to G_2 cell cycle arrest by neocarzinostatin (NCS). Exponentially growing normal (patient 1646) or SCAN1 (patient 1662) lymphoblasts were treated with 1 nM NCS for the indicated times and then cells were harvested, stained with propidium iodide and subjected to flow cytometry. Bar graphs show the cell cycle phase distributions as determined by ModFit software. Error bars represent the range of values obtained in two independent experiments. NCS-treated SCAN1 cells showed a larger G_2 fraction than normal cells at all time points, largely at the expense of S phase, which was severely depleted. By 48 hr, normal cells had nearly recovered an exponential profile, while G_2 arrest and S depletion persisted in SCAN1 cells, consistent with defective repair of NCS-induced DSBs.

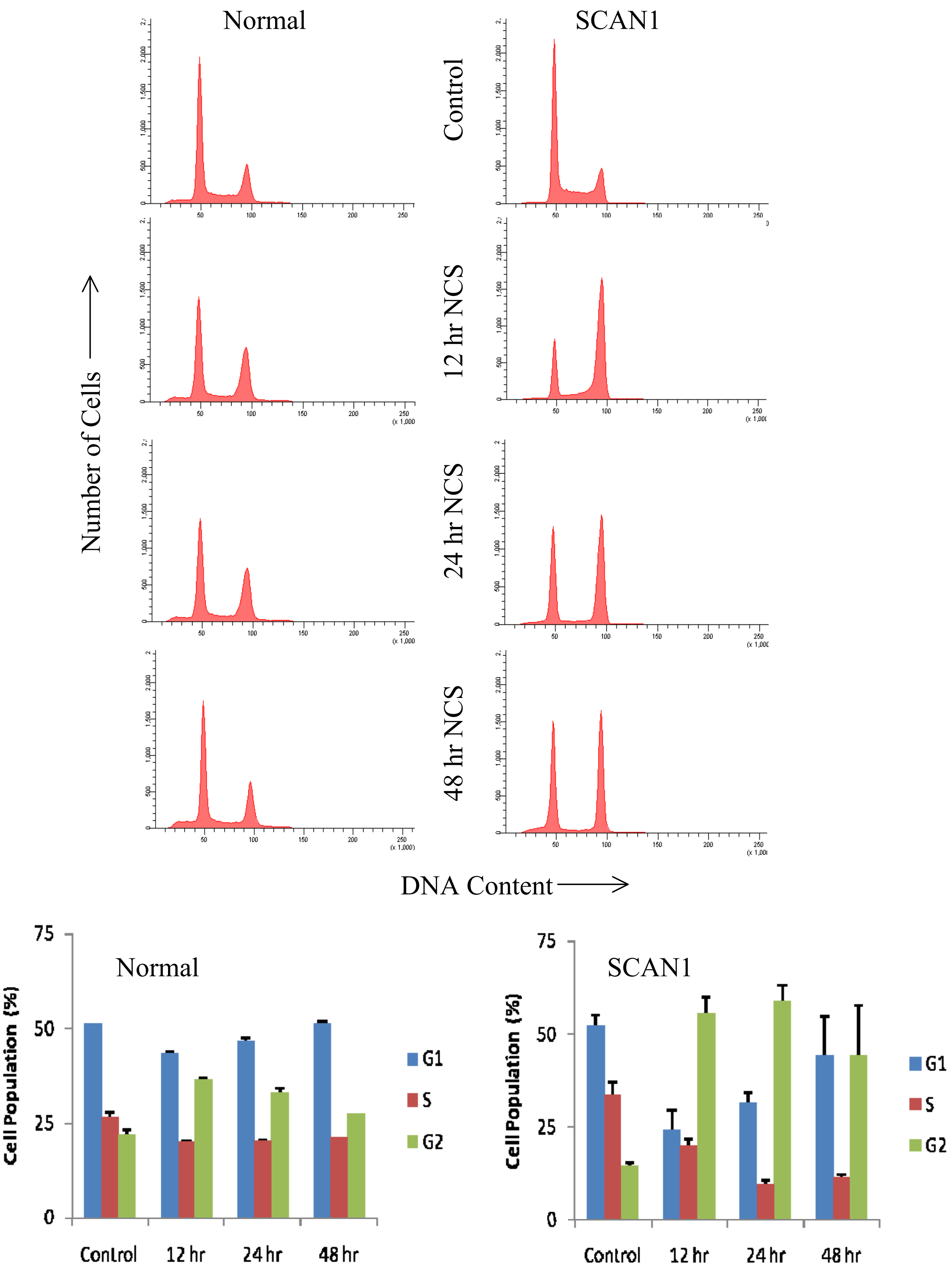
Normal
SCAN1
Control
12 hr NCS
24 hr NCS
48 hr NCS
Number of Cells
DNA Content
Normal
SCAN1
Cell Population (%)
G1
S
G2
Control
12 hr
24 hr
48 hr

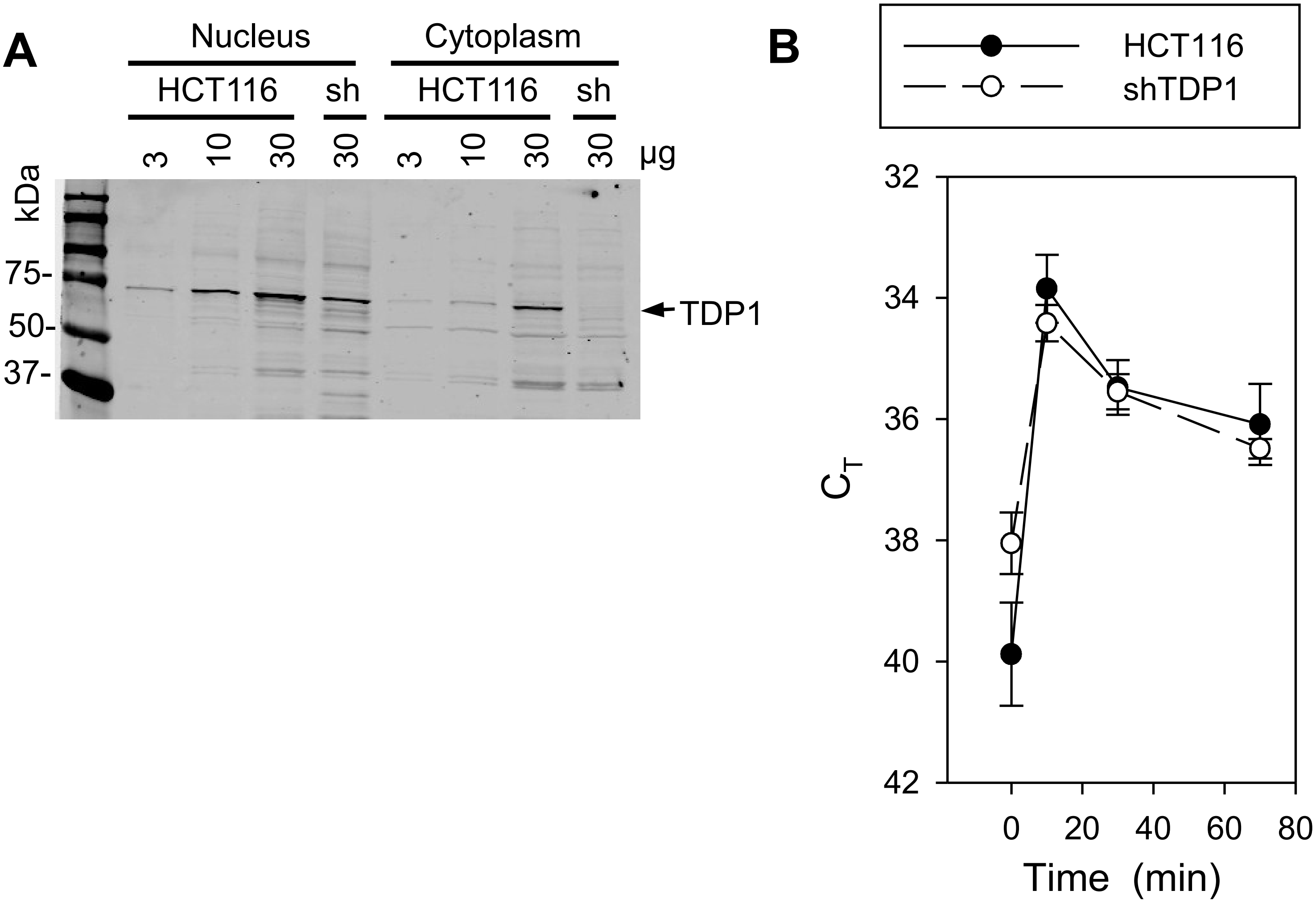

Supplemental Figure 9. Processing of 3′-PG DSB termini in shTDP1 knockdown cells. A. The extent of nuclear and cytoplasmic TDP1 knockdown was estimated by western blotting for cells stably expressing TDP1 shRNA from a lentiviral vector (sh), as compared to parental HCT116 colon carcinoma cells. TDP1 knockdown in cytoplasm was 90±5% (SEM, N=3) as determined by titration of TDP1 activity (Inamdar et al., 2002), but was too low to accurately estimate by densitometry. TDP1 knockdown in the nucleus was 68±3% by densitometry and 75±8% by titration of activity. B. Formation and removal of 3′-PG termini of NCS-C-induced DSBs in parental or shTDP1-knockdown HCT116 cells. Cells were treated with 5 µM NCS-C (as described for M059J cells in the Methods) and after various times 3′-PG termini were assessed by LMPCR (Mean ± SEM for 3 experiments). Knockdown of TDP1 had no apparent effect on 3′-PG processing in these cells. Simple siRNA transfection also did not produce a discernible effect on processing (data not shown).

Methods: The phosphorylated oligomers GATCCGGTGATAAGCGAGAGGCTAACTTCCTGTCATTA-GCCTCTCGCTTATCACTTTTTG and AATTCAAAAAGTGATAAGCGAGAGGCTAATGACAGGAA-GTTAGCCTGTCGCTTATGACCG were annealed and cloned into the BamHI and EcoRI sites of pLSLPw, and the vector was packaged into lentivirus (for details see Supplemental Material from Budanov et al., 2004). This vector expresses a hairpin that targets the sequence GUGAUAAGCGAGAGGCUA (bases 20300-20319 in exon 6 of the TDP1 gene, GenBank #NG009164). HCT116 cells were infected and cells grown for 1 day before selection for 4 days with 0.8 µg/ml puromycin. Cell lysates were prepared from individual puromycin-resistant clones and TDP1 activity was titrated using a 3′-phosphotyrosyl substrate (Inamdar et al., 2002) to select the lowest expressing clones. Cells below passage 12 were used for all experiments, and cytoplasmic and nuclear extracts (see Supplemental Fig. 7) for estimation of knockdown were prepared from the same cultures used for the LMPCR experiments. Proteins were resolved on 8% PAGE and blots were probed with a polyclonal mouse antibody to TDP1 (1:1000 dilution; Abnova #H00055775-A01). TDP1 levels were assessed by densitometry as in Supplemental Fig. 7, except that tubulin was used for normalization of both cytoplasmic and nuclear fractions.

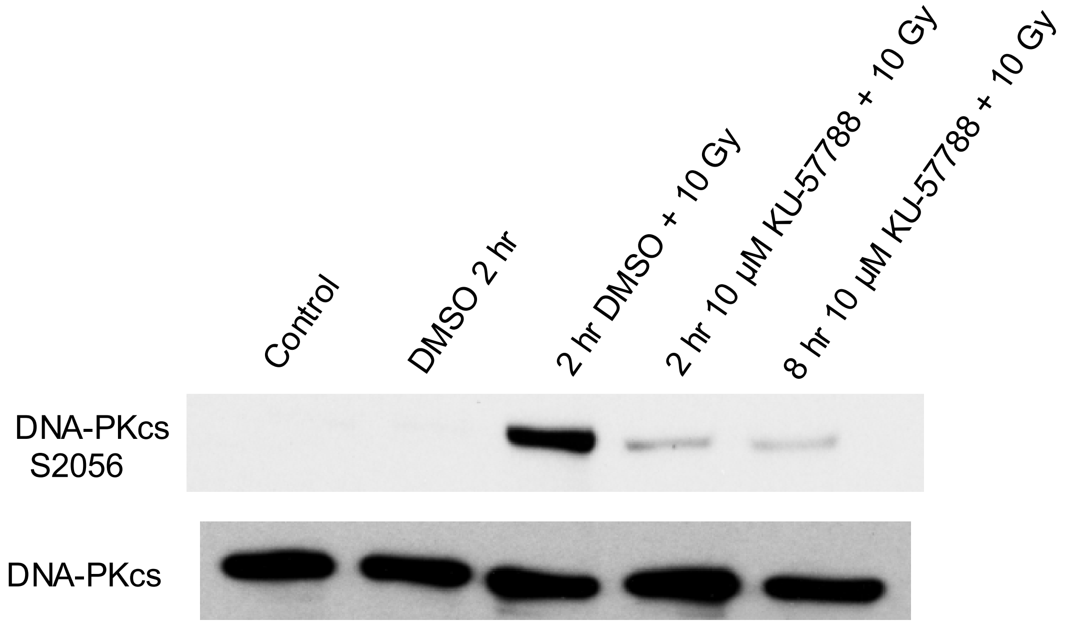

Supplemental Figure 10. Inhibition of DNA-PK catalytic activity by KU-57788 in normal lymphoblastoid cells as judged by autophosphorylation of serine 2056. Exponentially growing cells were treated with 10 µM KU-57788 (or 0.4 % DMSO, solvent for KU-57788) for 2 or 8 hr, exposed to 10 Gy ionizing radiation, and harvested 30 min later. Whole-cell lysates (30 µg/lane) were subjected to 5% PAGE, blotted onto nitrocellulose, and probed with a rabbit monoclonal antibody specific for phospho-S2056 of DNA-PK (Epitomics Cat# 3892-1, 1:1000). A duplicate blot was probed with a mouse monoclonal antibody against DNA-PKcs (BD Pharmingen Clone 4F10C5, Cat# 556456, 1000:1). Unlike many other DNA-PKcs phosphorylation sites, S2056 is phosphorylated only by DNA-PK and not by ATM (Chen et al., 2005). Thus, the dramatic reduction in the intensity of the phospho-S2056 band shows that KU-57788 treatment was highly effective in inhibiting DNA-PK activity, even though it did not significantly alter the formation or persistence of 3′-PG termini.

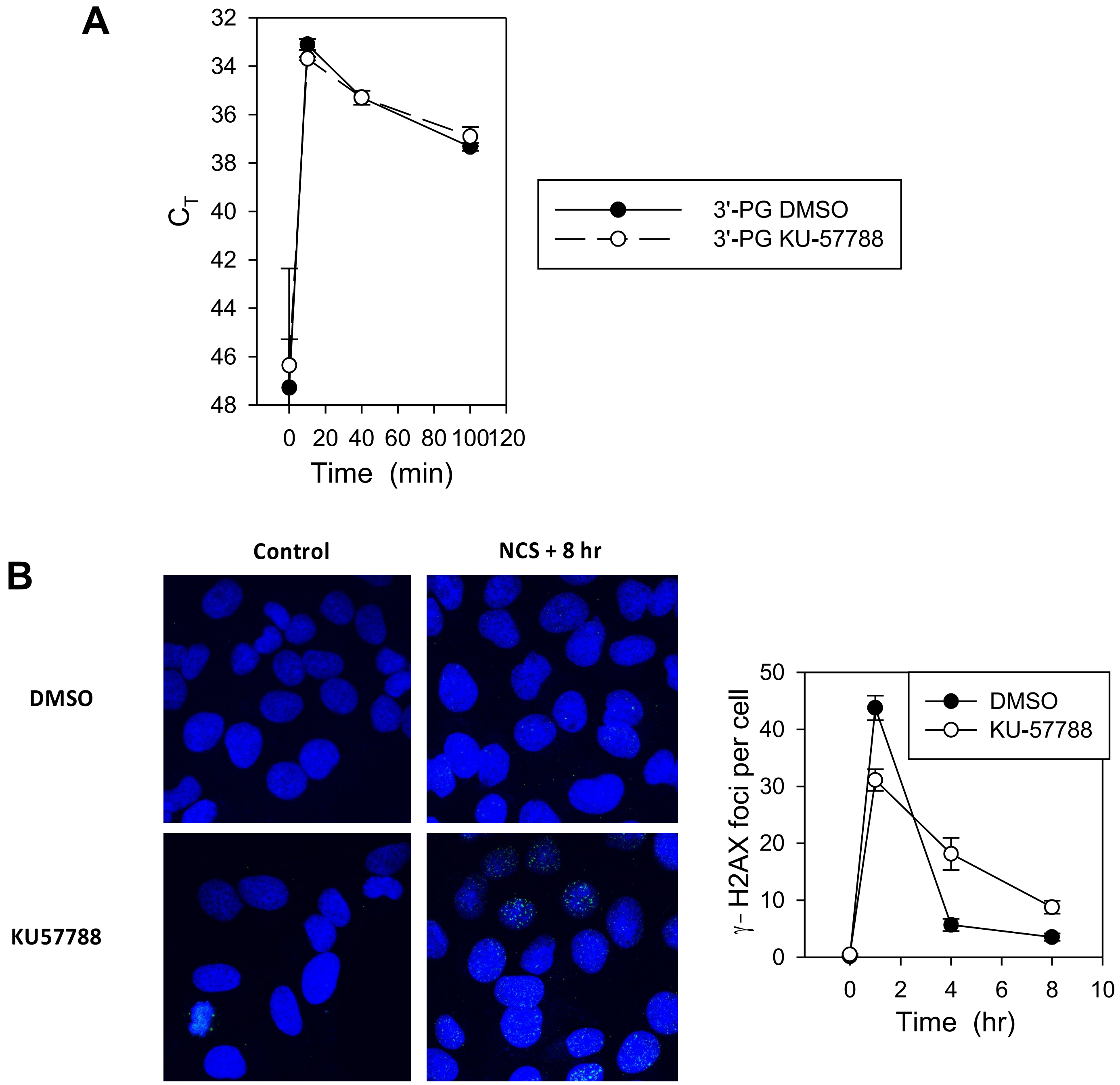

Supplemental Figure 11. Lack of effect of a DNA-PK inhibitor on 3′-PG processing despite inhibition of repair overall. (A) A549 lung carcinoma cells were incubated for 1 hr in medium containing 10 µM KU-57788 or vehicle (0.4% DMSO), trypsinized, washed, and treated with NCS-C in PBS for 10 min, then incubated in complete medium for 20 or 60 minutes to allow processing. DNA was isolated from the cells and subjected to chemical stabilization, enzymatic manipulations and ligation-mediated Taqman PCR for detection of DSB ends bearing 3′-PG termini. Error bars show SD for two Taqman PCR reactions with each sample. (B) Cells were plated on coverslips, incubated for 2 hr in the presence of 10 µM KU-57788 and treated (or not) with 6 nM NCS for 1 hr, incubated for 4 or 8 hr in the continued presence of KU-57788 to allow repair, and then stained for γ-H2AX foci (Mohapatra et al. 2011). Graph shows mean ± SEM for 25-50 cells for each data point. In both (A) and (B), KU-57788 was present throughout treatment, harvest and postincubation periods.

References for Supplemental Figures

Budanov, A.V., Sablina, A.A., Feinstein, E., Koonin, E.V. & Chumakov, P.M. (2004) Regeneration of peroxiredoxins by p53-regulated sestrins, homologs of bacterial AhpD. *Science* **304:** 596-600.

Chen B.P., Chan D.W., Kobayashi J., Burma S., Asaithamby A., Morotomi-Yano K., Botvinick, E., Qin, J. & Chen, D.J. (2005) Cell cycle dependence of DNA-dependent protein kinase phosphorylation in response to DNA double strand breaks. *J. Biol. Chem.* **280:** 14709-14715.

Freschauf, G.K., Karimi-Busheri, F., Ulaczyk-Lesanko, A., Mereniuk, T.R., Ahrens, A., Koshy, J.M., Rasouli-Nia, A., Pasarj, P., Holmes, C.F., Rininsland, F., Hall, D.G. & Weinfeld, M. (2009) Identification of a small molecule inhibitor of the human DNA repair enzyme polynucleotide kinase/phosphatase. *Cancer Res*. **69:** 7739-7746.

Inamdar,K.V., Pouliot,J.J., Zhou,T., Lees-Miller,S.P., Rasouli-Nia,A. and Povirk,L.F. (2002) Conversion of phosphoglycolate to phosphate termini on 3' overhangs of DNA double-strand breaks by the human tyrosyl-DNA phosphodiesterase hTdp1. *J. Biol. Chem.,* **276,** 24323-24330.

Mohapatra, S., Kawahara, M., Khan, I.S., Yannone, S.M. & Povirk, L.F. (2011) Restoration of G1 chemo/radioresistance and double-strand-break repair proficiency by wild-type but not endonuclease-deficient Artemis. *Nucleic Acids Res*. **39:** 6500-6510.

Rasouli-Nia, A., Karimi-Busheri, F. & Weinfeld, M. (2004) Stable down-regulation of human polynucleotide kinase enhances spontaneous mutation frequency and sensitizes cells to genotoxic agents. *Proc. Natl. Acad. Sci. USA* **101:** 6905-6910.

Schneider, T.D. & Stephens, R.M. (1990) Sequence logos: a new way to display consensus sequences. *Nucleic Acids Res.* **18:** 6097-6100.

Takashima, H., Boerkoel, C.F., John, J., Saifi, G.M., Salih, M.A., Armstrong, D., Mao, Y., Quiocho, F.A., Roa, B.B., Nakagawa, M., Stockton, D.W. & Lupski, J.R. (2002) Mutation of TDP1, encoding a topoisomerase I-dependent DNA damage repair enzyme, in spinocerebellar ataxia with axonal neuropathy. *Nature Genet.* **32:** 267-272.
